# Olopatadine plus mometasone for seasonal allergic rhinitis treatment: A pooled analysis of clinical trials

**DOI:** 10.1016/j.bjorl.2026.101817

**Published:** 2026-04-14

**Authors:** Marcio Nakanishi, Luis Felipe Barreiras Carbone, Vineet Aggarwal, Roger Hereward Gordonsmith, Wen Wu, Hanmant Barkate, Wilma Terezinha Anselmo-Lima

**Affiliations:** a Universidade de Brasília, Campus da Universidade Darcy Ribeiro, Faculdade de Medicina, Postgraduate Program in Medical Sciences, Asa Norte, DF, Brazil; b Global Medical Affairs, Glenmark Farmaceutica Ltda, São Paulo, SP, Brazil; c Global Medical Affairs, Glenmark Pharmaceuticals Ltda, Chakala, Off Western Express Highway, Andheri (E), Mumbai, India; d Global Medical Affairs, Glenmark Pharmaceuticals Europe Ltd, Unit 1 Building 2, Marlins Meadow, Watford, United Kingdom; e Universidade de São Paulo, Faculdade de Medicina de Ribeirão Preto, Department of Ophthalmology and Otorhinolaryngology, Ribeirão Preto, SP, Brazil

**Keywords:** Combination nasal spray, Mometasone furoate, Olopatadine hydrochloride, Pooled analysis, Seasonal allergic rhinitis

## Abstract

**Objective:**

This study was conducted to analyze the efficacy and safety of a fixed-dose combination of olopatadine HCl (antihistamine) and mometasone furoate (corticosteroid) (Olo/Mom, GSP301) for the treatment of Seasonal Allergic Rhinitis (SAR).

**Methods:**

Efficacy data from one phase II (NCT02318303 [GSP301-201]) and two pivotal phase III double-blind, randomized, active, placebo-controlled (NCT02631551 [GSP301-301] and NCT02870205 [GSP301-304]) clinical trials were collated and analyzed. These studies investigated Olo/Mom (administered twice daily/BID) compared with placebo and its monotherapy constituents for the treatment of SAR in patients aged ≥12-years. In addition, safety data were analyzed from the aforementioned studies and a proof-of-concept study (NCT03444506 [GSP301-POC]). Primary and secondary endpoints were compared between Olo/Mom, its active mometasone and olopatadine constituents, and placebo.

**Results:**

For the efficacy analyses (n = 2971), Olo/Mom significantly reduced rTNSS scores compared with placebo (Least Square Mean Difference [LSMD = −0.94]; 95% CI: −1.17, −0.70; p < 0.0001), olopatadine (LSMD = −0.37; 95% CI: −0.60, −0.14; p = 0.0019), and mometasone (LSMD = −0.42; 95% CI: −0.65, −0.18; p = 0.0005). Olo/Mom also significantly improved the RQLQ and iTNSS scores compared with placebo and its monotherapy constituents (p < 0.05, all). The onset of action of Olo/Mom was observed at 15-minutes and was sustained at subsequent timepoints. The incidence of treatment-emergent adverse events in the Olo/Mom, placebo, olopatadine, and mometasone groups was 13.9%, 9.5%, 13.2%, and 7.9%, respectively.

**Conclusion:**

This pooled analysis demonstrated the superiority of Olo/Mom compared to its monotherapy constituents or placebo in reducing SAR symptoms over 14-days. Moreover, this study showed that Olo/Mom has a rapid onset of action and significantly improves RQLQ scores compared to placebo. Administration of Olo/Mom BID is an effective and well-tolerated option for the treatment of SAR in patients aged 12-years or older.

**Level of evidence:**

I (Level I – randomized controlled trials).

## Highlights

• First pooled analysis of Olopatadine/Mometasone FDC.
• Reinforces significant improvements compared to mometasone and olopatadine.
• Compiled safety data demonstrate low AEs and high adherence to treatment.

## Introduction

Allergic Rhinitis (AR), characterized by nasal mucosal inflammation due to inhaled allergens,^1^ is categorized into Seasonal (SAR) or Perennial (PAR),^2^ with a global median prevalence of 18.1% (range: 1.0%–54.5%).^3^

Current management includes non-pharmacological measures and pharmacological interventions.[4], [5], [6], [7], [8] Treatment options include, Intranasal Antihistamines (INAH), such as olopatadine,^8^^,^^9^ and Intranasal Corticosteroids (INCS), such as mometasone furoate.^10^ Individually, administration of both drugs are effective and well-tolerated treatments for SAR.^11^ Recently updated guidelines recommend the use of INCS sprays or combined INAH/INCS sprays as first-line treatments for AR.^12^^,^^13^

The use of Fixed-Dose Combination (FDC) of olopatadine HCl and mometasone (GSP301, Olo/Mom) has been approved^14^ in many countries.[14], [15], [16] Clinical trials have demonstrated that Olo/Mom achieves significant and clinically meaningful improvements in the SAR symptoms than its individual components and placebo.^12^^,^^17^^,^^18^ Furthermore, previous studies suggest that FDCs may facilitate treatment adherence, which is often challenging when using two sprays.^19^

To support clinical and regulatory requirements, the efficacy data from three randomized clinical trials and safety data from an additional Proof-Of-Concept (POC) study were subjected to a pooled analysis.

## Methods

### Study design

The efficacy of Olo/Mom for the treatment of SAR was evaluated pooling data from three studies: one phase II (NCT02318303 [GSP301-201])^18^ and two pivotal phase III double-blind, randomized, active, placebo-controlled (NCT02631551 [GSP301-301]^11^ and NCT02870205 [GSP301-304])^17^ clinical trials. These studies, which were conducted in the US, have similar designs, populations, efficacy measures, and a 14-day treatment period. A 14-day treatment period is commonly used for assessing initial efficacy and safety and is consistent with standard practices in drug development. For the pooled safety assessment, data from an additional POC study conducted using a ragweed pollen environmental Exposure Chamber (EEC) model (NCT03444506 [GSP301-POC])^21^ was added to those obtained from the previously mentioned Phase II and III clinical trials. The details of these studies are summarized in Table S1.

The three efficacy studies included two phases: a 7–10 day placebo run-in period from screening to randomization, followed by a 15–17 day treatment phase from randomization to the final visit. After the placebo run-in, eligible patients were randomly assigned to receive one of four intranasal treatments for 14-days: Olo/Mom (665 μg olopatadine and 25 μg mometasone), olopatadine (665 μg; sponsor formulation), mometasone (25 μg; sponsor formulation), or placebo (GSP301 vehicle; contained inactive ingredients identical to the active treatments). The treatments were administered two sprays per nostril (BID) everyday over the treatment period.

### Study population

The three efficacy studies included males or non-pregnant females aged ≥12-years who were in good general health. The participants had a clinical history of SAR (at least 2 years before screening) with seasonal exacerbations and a documented positive skin prick test for relevant allergens. To qualify for enrollment, the participants were required to have a 12 -h reflective Total Nasal Symptom Score (rTNSS) of ≥8-points out of 12. Moreover, a congestion score ≥2 (of 3) was required for a.m. assessment at the screening visit.

Participants were excluded from the studies if they had not remained in a known pollen area for at least 24-hs during the last 7-days of the run-in period or for 2 consecutive days during the treatment period; used prohibited medications during the trials; had nasal or respiratory tract malformations, significant atopic dermatitis or rhinitis medicamentosa, or any upper or lower respiratory infection, including acute or chronic otitis media and rhinosinusitis within 14-days before randomization.

### Efficacy measurements

#### Primary endpoint

The primary efficacy endpoint in each of the three efficacy studies was the change from the average self-reported a.m. and p.m. 12-h rTNSS baseline over the 14-day treatment period. The rTNSS is the sum of four nasal symptom scores (congestion, rhinorrhea, itching and sneezing), each rated from 0 (none) to 3 (severe). Thus, the total rTNSS ranges from 0 to 12.

#### Secondary endpoints

Key secondary efficacy endpoints included change in the baseline average a.m. and p.m. 12-h instantaneous TNSS (iTNSS) over 14-days and change in the average a.m. and p.m. reflective total ocular symptom score (rTOSS; sum of 0–3 points each for itchy eyes, watery eyes, and ocular redness) over 14 days. Additional outcomes included the onset of action on Day 1 (time to significant symptom relief vs. placebo), the overall change in the baseline Rhinoconjunctivitis Quality of Life Questionnaire (standardized version) (RQLQ) score by Day 15, and the durability of the improvement in rTNSS over the 14-day treatment period.

Drug compliance and other baseline characteristics were assessed in these studies. Patients who took 75%–125% of the expected doses were considered compliant.

### Safety assessment

Data on safety profiles, including Treatment-Emergent Adverse Events (TEAEs), Serious Adverse Events (SAEs), clinical laboratory measurements, and physical examinations, were pooled and presented for treatment groups across the four studies.

### Statistical analysis

The datasets were set up using the Clinical Data Interchange Consortium Study Data Tabulation Model v3.1.3 and the Analysis Dataset Model v2.1. Statistical analyses were conducted using SAS® v9.4, with Full Analysis Set (FAS) as the primary analysis population. A Mixed-effect Model for Repeated Measures analysis (MMRM) was performed to assess the primary and secondary efficacy endpoints. The adjusted mean (Least-Squares [LS] mean) change in baseline values over the 14-day treatment period, including treatment differences (95% Confidence Intervals [95% CIs] and p-values), is presented for each treatment group. Sensitivity analysis of the primary efficacy endpoint was performed as a secondary analysis using an Analysis of Covariance (ANCOVA) model. Descriptive statistics were used to summarize the baseline, post-treatment, and change from baseline in the average a.m. and p.m. rTNSS, iTNSS, and rTOSS values according to treatment group and day. The ANCOVA model was used to evaluate the changes in RQLQscores. Subgroup analysis was performed to examine the effectiveness of Olo/Mom according to age, sex, race, and ethnicity. TEAEs were coded for clinical safety according to the Medical Dictionary for Regulatory Activities (MedDRA) v20.0.

## Results

### Patient population

**Fig. 1:**
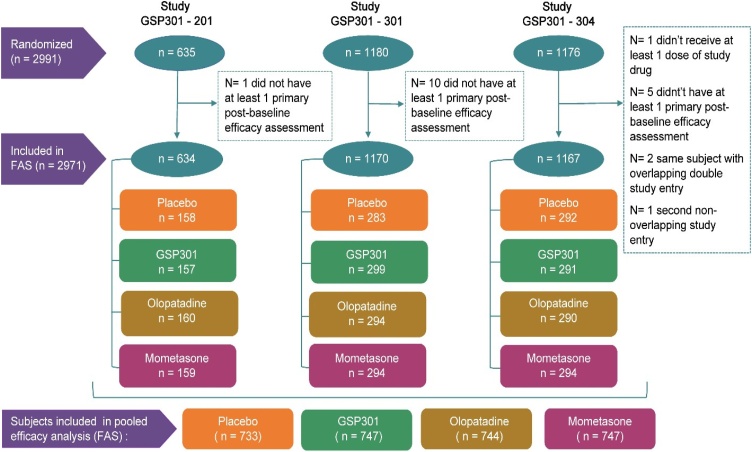
Patient selection flow diagram. FAS, Full Analysis Set.

Of 4912 eligible participants screened across the efficacy studies, 4477 entered the run-in period, and 2991 randomized participants were included in the integrated efficacy dataset. The FAS included 2971 participants who met the defined parameters for analysis (Fig. 1).

Baseline demographic and physical characteristics were similar across the treatment groups (Table 1), with mean age of 40.2-years, and body mass index of 30.13 kg/m^2^. Treatment compliance was 94% or more in all treatment groups.

**Table 1:** Demographic data, physical characteristics, and treatment compliance data.

		Number (%) of participants				
		Placebo (n = 733)	GSP301 (Olo/Mom) (n = 747)	Olopatadine (n = 744)	Mometasone (n = 747)	Total (n = 2971)
Age (years) (mean ± SD)		40.5 ± 15.08	40.2 ± 15.02	40.3 ± 14.63	39.9 ± 15.36	40.2 ± 15.02
Age group (years), n (%)^a^	12–17	62 (8.5)	65 (8.7)	54 (7.3)	64 (8.6)	245 (8.2)
	18–64	635 (86.6)	649 (86.9)	658 (88.4)	639 (85.5)	2581 (86.9)
	≥65	36 (4.9)	33 (4.4)	32 (4.3)	44 (5.9)	145 (4.9)
Sex, n (%)^a^	Male	267 (36.4)	235 (31.5)	277 (37.2)	283 (37.9)	1062 (35.7)
	Female	466 (63.6)	512 (68.5)	467 (62.8)	464 (62.1)	1909 (64.3)
Race, n (%)^a^	White	590 (80.5)	617 (82.6)	591 (79.4)	583 (78.0)	2381 (80.1)
	Black/African American	129 (17.6)	108 (14.5)	130 (17.5)	136 (18.2)	503 (16.9)
	Other	14 (1.9)	22 (2.9)	23 (3.1)	28 (3.7)	87 (2.9)
Ethnicity, n (%)^a^	Hispanic or Latino	202 (27.6)	218 (29.2)	235 (31.6)	224 (30.0)	879 (29.6)
	Not Hispanic or Latino	531 (72.4)	529 (70.8)	509 (68.4)	523 (70.0)	2092 (70.4)
Height (m) (mean ± SD)		1.67 ± 0.101	1.67 ± 0.102	1.67 ± 0.101	1.68 ± 0.102	1.67 ± 0.102
Weight (kg) (mean ± SD)		83.92 ± 23.444	83.56 ± 22.001	85.47 ± 23.850	84.12 ± 22.084	84.27 ± 22.855
BMI (kg/m^2^) (mean ± SD)		30.04 ± 9.233	30.09 ± 8.684	30.42 ± 7.850	29.97 ± 8.576	30.13 ± 8.595
Treatment compliance, n (%)^a^, ^b^	<75%	5 (0.7)	2 (0.3)	4 (0.5)	5 (0.7)	16 (0.5)
	>75% ‒ ≤125%	691 (94.3)	720 (96.3)	709 (95.3)	702 (94.0)	2822 (95.0)
	>125%	37 (5.0)	25 (3.3)	31 (4.2)	40 (5.4)	133 (4.5)

BMI, Body Mass Index; max, Maximum; min, Minimum; N, Number of participants in the treatment group; n, Number of participants with data available; SD, Standard Deviation.

a Percentages are based on the total number of participants in each treatment group.

b Treatment compliance: (total number of doses actually taken/total number of doses expected) ×100.

### Efficacy assessments

#### Primary efficacy measurements

**Fig. 2:**
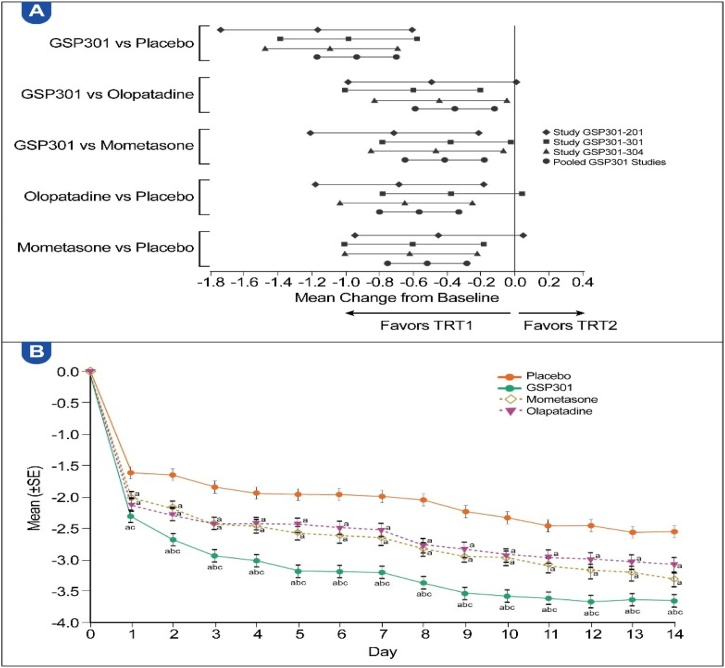
(A) Differences in mean score change from baseline to day 14, LS means with 95% CIs of average a.m. and p.m. rTNSS (individual studies and all pooled participants, full analysis set). (B) LS means of change in average a.m. and p.m. rTNSS for each day (all pooled participants, full analysis set). NS, nasal spray; SE, standard error; TRT, treatment. ^a^ Statistically significant difference (p < 0.05) vs. placebo. ^b^ Statistically significant difference (p < 0.05) vs. olopatadine. ^c^ Statistically significant difference (p < 0.05) vs. mometasone.

The pooled efficacy analysis over the 14-day treatment period showed that Olo/Mom was more effective than placebo and the monotherapies. The LS mean differences (LSMD) of Olo/Mom compared with placebo (−0.94 [95% CI: −1.17, −0.70; p < 0.0001]), olopatadine (−0.37 [95% CI: −0.60, −0.14; p = 0.0019]), and mometasone (−0.42 [95% CI: −0.65, −0.18; p = 0.0005]) were clinically meaningful and statistically significant from Day 1 to Day 14 (Table 2). Sensitivity analysis confirmed the primary MMRM results (Table S2). Fig. 2A shows a forest plot of the individual and pooled studies, whereas Fig. 2B illustrates the daily improvements in the average a.m. and p.m. rTNSS of the Olo/Mom group versus the placebo group. The average a.m. and p.m. rTNSS results are summarized in Tables S3 and S4.

**Table 2:** Results of the MMRM analysis of average a.m. and p.m. rTNSS over the 14-day treatment period (all pooled participants, full analysis set).

Treatment group comparisons (TRT1 vs. TRT2)	n		LS mean		Comparison between TRT1 and TRT2		
	TRT1	TRT2	TRT1	TRT2	LS mean difference (SE)	95% CI	p-value
GSP301 vs. placebo	747	731	−3.30	−2.36	−0.94 (0.120)	(−1.17, −0.70)	<0.0001^a^
GSP301 vs. olopatadine	747	744	−3.30	−2.93	−0.37 (0.119)	(−0.60, −0.14)	0.0019^a^
GSP301 vs. mometasone	747	746	−3.30	−2.89	−0.42 (0.119)	(−0.65, −0.18)	0.0005^a^
Olopatadine vs. placebo	744	731	−2.93	−2.36	−0.57 (0.119)	(−0.80, −0.33)	<0.0001^a^
Mometasone vs. placebo	746	731	−2.89	−2.36	−0.52 (0.119)	(−0.75, −0.29)	<0.0001^a^

a.m., Morning; CI, Confidence Interval; LS, Least-Squares; MMRM, Mixed-effect Model for Repeated Measures; n, Number of participants with data available; p.m., evening; rTNSS, reflective Total Nasal Symptom Score; SE, Standard Error; TRT, Treatment.

Statistical analysis model: MMRM model with change from baseline set as the dependent variable; treatment group, study, and site as fixed effects; baseline score as the covariate; and study day as the within-subject effect. The variance-covariance matrix used is unstructured.

a Statistically significant difference (p < 0.05).

#### Primary efficacy endpoint (rTNSS)

Over the 14-day treatment period, Olo/Mom achieved greater improvements in nasal symptom scores than the other treatments. The average a.m./p.m. rTNSS showed that Olo/Mom was significantly more effective than placebo and either of its monotherapy components (Table 2). The LSMD (Olo/Mom minus comparator) change in rTNSS for Olo/Mom was −0.94 (95% CI: −1.17 to −0.70; p < 0.0001), −0.37 (95% CI: −0.60 to −0.14; p = 0.0019) and −0.42 (95% CI: −0.65 to −0.18; p = 0.0005), versus placebo, olopatadine, and mometasone, respectively. These differences were both statistically and clinically meaningful, in line with the treatment effects observed in the individual trials. Superiority of Olo/Mom was apparent from Day 1 and persisted until Day 14 (Fig. 2B). A forest plot of the treatment effect reported in each study and the pooled analysis of the treatment effects (Fig. 2A) indicated consistent efficacy, favoring Olo/Mom, across studies. Additional sensitivity analysis are consistent with those of the MMRM, confirming the robustness of the primary analysis (Table S2).

#### Secondary efficacy endpoints

##### Instantaneous TNSS (iTNSS)

**Fig. 3:**
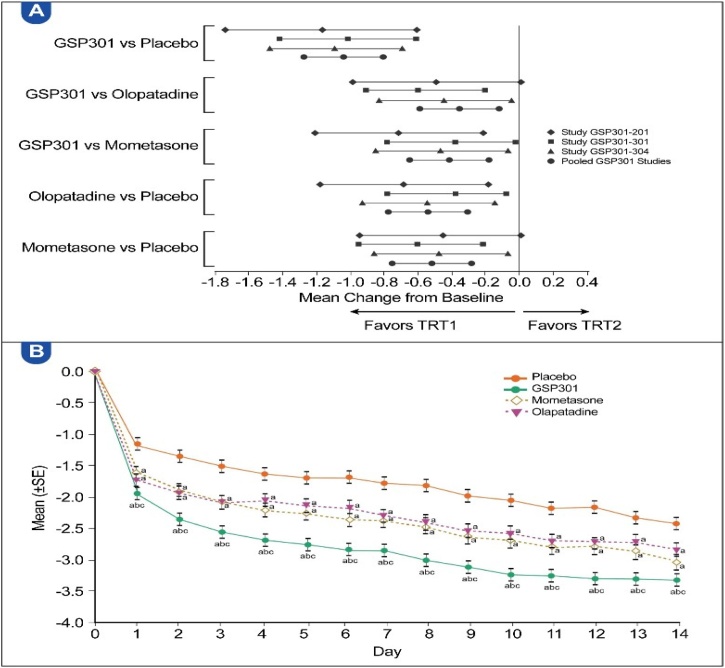
(A) Differences in mean score change from baseline to day 14, LS means with 95% CIs of average a.m. and p.m. iTNSS (individual studies and all pooled participants, full analysis set). (B) LS means of change in average a.m. and p.m. iTNSS for each day (all pooled participants, full analysis set). NS, Nasal Spray; SE, Standard Error; TRT, Treatment. ^a^ Statistically significant difference (p < 0.05) vs. placebo. ^b^ Statistically significant difference (p < 0.05) vs. olopatadine. ^c^ Statistically significant difference (p < 0.05) vs. mometasone.

Over the 14-day treatment period, Olo/Mom showed superior efficacy as reflected by the mean change in the baseline average a.m. and p.m. iTNSS compared with the placebo and monotherapies. The LSMD for Olo/Mom was −0.91 (95% CI: −1.14, −0.69; p < 0.0001), −0.37 (95% CI: −0.59, −0.16; p = 0.0008), and −0.44 (95% CI: −0.65, −0.22; p = 0.0001) versus placebo, olopatadine and mometasone, respectively (Table 3). Fig. 3A shows a forest plot of the individual and pooled studies, whereas Fig. 3B indicates daily improvements in the average a.m. and p.m. iTNSS for the Olo/Mom versus placebo groups.

**Table 3:** Results of the repeated measures analysis of the average a.m. and p.m. iTNSS over the 14-day treatment period (all pooled participants, full analysis set).

Treatment group comparison (TRT1 vs. TRT2)	n		LS mean		Comparison between TRT1 and TRT2		
	TRT1	TRT2	TRT1	TRT2	LS mean difference (SE)	95% CI	p-value
GSP301 vs. placebo	747	731	−2.94	−2.02	−0.91 (0.113)	(−1.14, −0.69)	<0.0001^a^
GSP301 vs. olopatadine	747	744	−2.94	−2.56	−0.37 (0.112)	(−0.59, −0.16)	0.0008^a^
GSP301 vs. mometasone	747	746	−2.94	−2.50	−0.44 (0.112)	(−0.65, −0.22)	0.0001^a^
Olopatadine vs. placebo	744	731	−2.56	−2.02	−0.54 (0.112)	(−0.76, −0.32)	<0.0001^a^
Mometasone vs. placebo	746	731	−2.50	−2.02	−0.48 (0.112)	(−0.70, −0.26)	<0.0001^a^

a.m., Morning; CI, Confidence Interval; iTNSS, instantaneous Total Nasal Symptom Score; LS, Least-Squares; n, Number of participants with data available; p.m., Evening; SE, Standard Error; TRT, Treatment.

Statistical analysis model: Mixed-effect model for repeated measures with change from baseline set as the dependent variable; treatment group, study, and site as fixed effects; baseline score as the covariate; and study day as the within-subject effect. The variance-covariance matrix used is unstructured.

a Statistically significant difference (p < 0.05).

##### Reflective Total Ocular Symptom score (rTOSS)

**Fig. 4:**
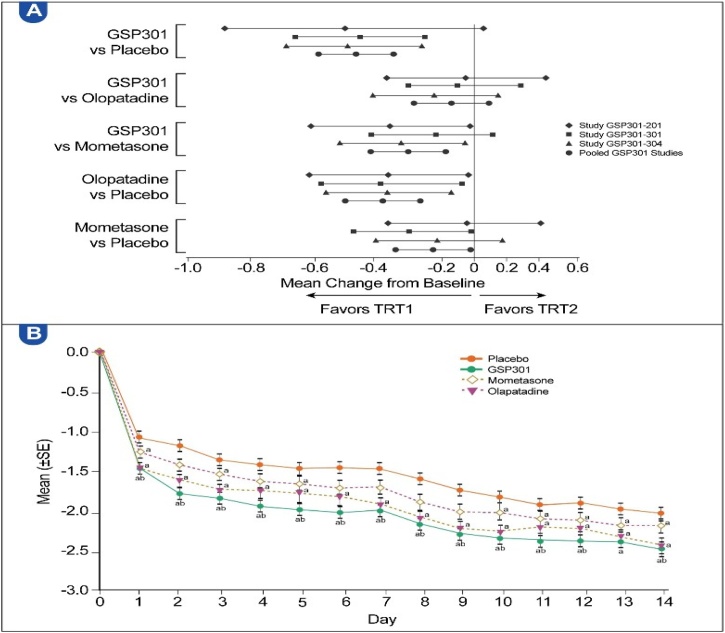
(A) Differences in mean score change from baseline to day 14, LS means with 95% CIs of average a.m. and p.m. rTOSS (individual studies and all pooled participants, full analysis set). (B) LS means of change in average a.m. and p.m. rTOSS for each day (all pooled participants, full analysis set). NS, Nasal Spray; SE, Standard Error; TRT, treatment. ^a^ Statistically significant difference (p < 0.05) vs. placebo. ^b^ Statistically significant difference (p < 0.05) vs. mometasone.

Olo/Mom achieved a significantly lower LS mean (−2.18) than placebo (−1.71) over 14-days (LSMD = −0.47; 95% CI, −0.66, −0.28; p < 0.0001). Although no significant difference between Olo/Mom and olopatadine was observed, Olo/Mom demonstrated statistically significant improvements compared with mometasone (Table 4). Fig. 4A presents a forest plot of the results for the individual and pooled studies, whereas Fig. 4B shows the daily improvement in the average a.m. and p.m. rTOSS for the Olo/Mom versus placebo.

**Table 4:** Results of the repeated measures analysis of the average a.m. and p.m. rTOSS over the 14-day treatment period (all pooled participants, full analysis set).

Treatment group comparison (TRT1 vs. TRT2)	n		LS mean		Comparison between TRT1 and TRT2		
	TRT1	TRT2	TRT1	TRT2	LS mean difference (SE)	95% CI	p-value
GSP301 vs. placebo	747	731	−2.18	−1.71	−0.47 (0.095)	(−0.66, −0.28)	<0.0001^a^
GSP301 vs. olopatadine	747	744	−2.18	−2.08	−0.10 (0.094)	(−0.28, 0.09)	0.3045
GSP301 vs. mometasone	747	746	−2.18	−1.90	−0.27 (0.094)	(−0.46, −0.09)	0.0037^a^
Olopatadine vs. placebo	744	731	−2.08	−1.71	−0.37 (0.095)	(−0.56, −0.18)	<0.0001^a^
Mometasone vs. placebo	746	731	−1.90	−1.71	−0.19 (0.095)	(−0.38, −0.01)	0.0410^a^

a.m., Morning; CI, Confidence Interval; LS, Least-Squares; n, Number of participants with data available; p.m., Evening; rTOSS, reflective Total Ocular Symptom Score; SE, Standard Error; TRT, Treatment.

Statistical analysis model: Mixed-effect model for repeated measures with change from baseline set as the dependent variable; treatment group, study, and site as fixed effects; baseline score as the covariate; and study day as the within-subject effect. The variance-covariance matrix used is unstructured.

a Statistically significant difference (p < 0.05).

##### Rhinoconjunctivitis Quality of Life Questionnaire (RQLQ)

Statistically significant improvement in overall RQLQ scores were noted for Olo/Mom at the end of the treatment period compared to the placebo (LSMD = −0.48; 95% CI: −0.67, −0.30; p < 0.0001), olopatadine (LSMD = −0.29; 95% CI: −0.47, −0.11; p < 0.0001), and mometasone (LSMD = −0.19; 95% CI: −0.37, −0.01; p = 0.0058) (Table 5).

**Table 5:** ANCOVA of the overall RQLQ(S) scores on day 15 (all pooled participants, full analysis set).

Treatment group comparison (TRT 1 vs. TRT 2)	n		LS mean		Comparison between TRT 1 and TRT 2		
	TRT 1	TRT 2	TRT 1	TRT 2	LS mean difference (SE)	95% CI	p-value
GSP301 vs. placebo	725	707	−1.60	−1.12	−0.48 (0.071)	(−0.67, −0.30)	<0.0001^a^
GSP301 vs. olopatadine	725	721	−1.60	−1.31	−0.29 (0.070)	(−0.47, −0.11)	<0.0001^a^
GSP301 vs. mometasone	725	724	−1.60	−1.41	−0.19 (0.070)	(−0.37, −0.01)	0.0058^a^
Olopatadine vs. placebo	721	707	−1.31	−1.12	−0.19 (0.071)	(−0.37, −0.01)	0.0070^a^
Mometasone vs. placebo	724	707	−1.41	−1.12	−0.29 (0.071)	(−0.47, −0.11)	<0.0001^a^

ANCOVA, Analysis of Covariance; CI, Confidence Interval; LS, Least-Squares; n, Number of participants with data available; p.m., Evening; RQLQ(S), Rhinoconjunctivitis Quality of Life Questionnaire (standardized); SE, Standard Error; TRT, Treatment.

Baseline is defined as the pre-dosing timepoint at the randomization visit (Visit 2).

Statistical analysis model: ANCOVA with change from baseline set as the dependent variable; treatment group, study, and site as fixed effects; and baseline as the covariate.

For study GSP301-201, RQLQ(S) data were not collected for 12–17-year-old participants, in accordance with the protocol.

a Statistically significant difference (p < 0.05).

Significant improvement in all domain scores (activities, emotional, eye symptoms, nasal symptoms, non-nose/eye symptoms, practical problems, and sleep) was observed compared to the placebo group (Table S5). Additionally, statistically significant differences favoring Olo/Mom compared with olopatadine and mometasone were observed for all domain scores at Day 15, with the exception of the sleep domain comparison with mometasone.

##### Onset of action

**Fig. 5:**
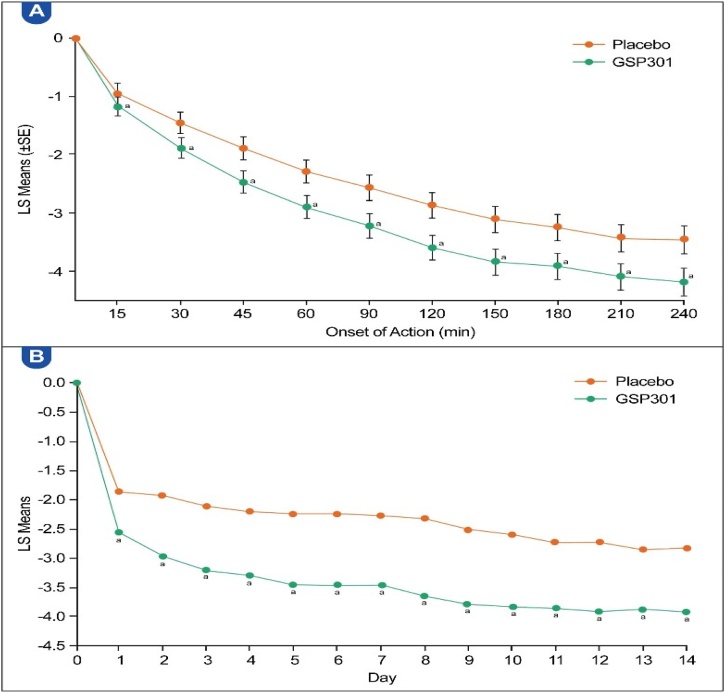
(A) LS means of change in onset of action over the 4-h assessment period (all pooled participants, full analysis set). (B) Durability of the improvement in rTNSS (all pooled participants, full analysis set). SE, Standard Error; TRT, Treatment; LS, Least Square. ^a^ Statistically significant difference (p < 0.05) vs. placebo.

The time to onset of action for Olo/Mom was evident at 15-minutes after the first dose, LSMD of Olo/Mom compared with the placebo was 0.23 (95% CI: −0.41, −0.05; p = 0.0110). The statistically significant LSMD between Olo/Mom and the placebo persisted for up to 4 h post-dosing, demonstrating sustained effectiveness (Fig. 5A). The onset of action for olopatadine was observed at 30-minutes post-dose, whereas that for mometasone could not be confirmed, as only one significant difference (at 240 min) was noted (Table S6).

##### Durability of response

The symptom relief achieved with Olo/Mom was maintained over the 14-day treatment period, without evidence of tachyphylaxis. Improvement in rTNSS with Olo/Mom versus placebo was sustained each day, and repeated-measures analysis confirmed a significant treatment-by-time benefit (p < 0.0001) (Fig. 5B).

### Comparison of subgroup results

The efficacy of Olo/Mom was consistently observed across demographic subgroups. Efficacy outcomes for Olo/Mom were favorable compared to placebo, regardless of age group, sex, race, or ethnicity, with no evidence of any subgroup deriving disproportionally different benefits (Table S7). Thus, the benefits of Olo/Mom appeared to be consistent across the entire patient population.

### Safety results

The incidence of TEAEs in the Olo/Mom group was 13.9%, whereas that in the placebo, olopatadine, and mometasone groups was 9.5%, 13.2%, and 7.9%, respectively (Table 6). Related TEAEs occurred in 6.5% of the patients in the Olo/Mom group versus 2.6%, 6.0%, and 2.7% in the placebo, olopatadine, and mometasone groups, respectively. The incidences of dysgeusia, epistaxis, and nasal discomfort in the Olo/Mom group were higher than those in the placebo group. Seventeen participants (0.6%) discontinued treatment for Olo/Mom due to TEAEs. The incidences of TEAEs in the Olo/Mom (0.4%) and placebo (0.1%) groups were low, and similar rates were observed in the monotherapy groups (0.9% for olopatadine and 0.8% for mometasone).

**Table 6:** Total number of TEAEs, their severity, relationship to the study drug, and their distribution across treatment groups in the pooled safety analysis of GSP301-POC, GSP301-201, GSP301-301, and GSP301-304.

Participant-level summary	Placebo (n = 776)^a^	GSP301 (n = 789)^a^	Olopatadine HCl (n = 751)^a^	Mometasone (n = 746)^a^	Total (n = 3062)
Participants with at least one TEAE, n (%)^b^	74 (9.5)	110 (13.9)	99 (13.2)	59 (7.9)	342 (11.2)
Most frequently reported (≥1% of participants in any treatment group or overall) TEAEs					
Nervous system disorders					
Dysgeusia^c^	2 (0.3)	24 (3.0)	16 (2.1)	0	42 (1.4)
Headache	14 (1.8)	6 (0.8)	8 (1.1)	7 (0.9)	35 (1.1)
Respiratory, thoracic and mediastinal disorders					
Epistaxis	5 (0.6)	8 (1.0)	11 (1.5)	6 (0.8)	30 (1.0)
Nasal discomfort	6 (0.8)	8 (1.0)	4 (0.5)	4 (0.5)	22 (0.7)
Others (<1%)	47 (6.0)	64 (8.1)	60 (8.0)	42 (5.7)	213 (7.0)
Severity					
Mild	45 (5.8)	65 (8.2)	54 (7.2)	29 (3.9)	193 (6.3)
Moderate	25 (3.2)	41 (5.2)	34 (4.5)	24 (3.2)	124 (4.0)
Severe	4 (0.5)	4 (0.5)	11 (1.5)	6 (0.8)	25 (0.8)
Participants with at least one TEAE that led to discontinuation of the study drug, n (%)	1 (0.1)	3 (0.4)	7 (0.9)	6 (0.8)	17 (0.6)
Participants with at least one related TEAE^c^, n (%)	20 (2.6)	51 (6.5)	45 (6.0)	20 (2.7)	136 (4.4)
Participants with at least one severe TEAE, n (%)	4 (0.5)	4 (0.5)	11 (1.5)	6 (0.8)	25 (0.8)
Participants with SAE, n (%)	1 (0.1)	1 (0.1)	2 (0.3)	1 (0.1)	5 (0.2)
Participants with severe SAE, n (%)	1 (0.1)	0	2 (0.3)	0	3 (0.1)
Severity					
Moderate	0	1 (0.1)	0	1 (0.1)	2 (0.1)
Severe	1 (0.1)	0	2 (0.3)	0	3 (0.1)
Participants with at least one SAE that led to discontinuation of the study drug, n (%)	1 (0.1)	0	0	0	1 (0.0)

a N, total number of participants in each treatment group in the safety analysis set; n, number of participants with adverse events.

b Percentages in the succeeding categories are based on total number of participants (for participant-level summary) OR events (for event-level summary) in the SAS within each treatment group. Participants with multiple events in the same category were counted only once in that category. Participants with events in more than one category were counted once in each of those categories.

c Adverse events were classified as 'Related' to the study treatment if the relationship was categorized as Possible, Probable, or Definite according to the CRF. Missing relationship to the study drug was considered as related.

No significant differences in SAEs were observed between the Olo/Mom and placebo groups, or either of the monotherapy groups. No SAE was considered related to Olo/Mom, and no death was reported. (Tables S8 and S10). TEAEs that led to treatment discontinuation are shown in Table S9. No differences in laboratory values, vital signs, physical findings, ECGs, or ENT findings were observed between Olo/Mom and placebo or monotherapy groups.

## Discussion

Combining an INAH with an INCS in a single spray provides superior symptom control compared to monotherapies,^20^^,^^21^ potentially lowering medical expenses,^22^ improving adherence, and yielding considerable benefits over co-administered monocomponents.[23], [24], [25] The efficacy and safety of Olo/Mom have been studied in patients with AR, including patients with SAR,^11^^,^^17^^,^^18^ pediatric patients,^24^ and patients with PAR.^25^ A 52-week study (GSP301-303) demonstrated sustained efficacy of Olo/Mom versus placebo for the treatment of PAR, corroborating findings observed in SAR populations.^25^

### Olo/Mom versus placebo

In this pooled analysis, Olo/Mom alleviated nasal and ocular symptoms (indicated by significant improvement in rTNSS, iTNSS, and rTOSS) and enhanced quality of life, (indicated by significant improvement in RQLQ scores), compared to placebo (all p < 0.0001). Although the difference in rTOSS in the phase II study did not reach statistical significance,^18^ both phase III trials confirmed statistical superiority of Olo/Mom over placebo.^11^^,^^17^

### Olo/Mom versus olopatadine and mometasone

This study showed that Olo/Mom significantly improved rTNSS, iTNSS, and RQLQ score compared to olopatadine and mometasone. The improvements in rTNSS compared to mometasone in the GSP301-301 study and in iTNSS compared to olopatadine in the GSP301-201 study were clinically meaningful but not statistically significant (p = 0.0587 and 0.0584, respectively).^11^^,^^18^ This pooled analysis and the individual studies, except for GSP301-301, demonstrated that Olo/Mom significantly improved rTOSS compared to mometasone.^11^ Additionally, Olo/Mom improved rTOSS compared with olopatadine (LSMD = −0.10; p = 0.3045); however, this result was not statistically significant. This finding is not unexpected because antihistamines (olopatadine) are more efficacious than corticosteroids (mometasone) in improving the ocular symptoms of SAR.^26^^,^^27^ Regarding RQLQ, while the pooled results were significant, the results for GSP301-201 (versus olopatadine) and GSP301-304 (versus mometasone) did not reach statistical significance.^17^^,^^18^

### Olopatadine and mometasone versus placebo

In pooled analysis, both olopatadine nasal spray and mometasone nasal spray were significantly more effective than placebo in achieving primary and most secondary endpoints, supporting their efficacy in the treatment SAR. Overall, neither monotherapy produced improvements as broad or consistent as those achieved with the combination therapy.

### Onset of action

Patients with AR seek treatments that provide effective, prompt, and sustained relief.^11^ In this pooled analysis and in the phase III studies (GSP301-301 and GSP301-304),^11^^,^^17^ Olo/Mom showed a rapid onset of action (15-minutes after first dose). In an EEC study, change in iTNSS from baseline was statistically significant at 10-minutes and was maintained at 11 of 12 timepoints across 4 hours.^28^

### Safety and tolerability

The safety profile of Olo/Mom observed in this pooled analysis is consistent with those reported in individual trials. The types of AEs observed (e.g., mild nasal irritation, unpleasant taste, minor nosebleeds) are typical for INCS/INAH combinations and no new safety concerns or synergistic adverse effects were identified. The slightly higher incidence of certain local AEs, such as dysgeusia, with Olo/Mom (compared to the placebo or mometasone group), can be attributed to the olopatadine component. This side effect, although bothersome to a few, can often be managed (e.g., by using a proper spray technique and not inhaling too deeply to avoid throat runoff) and did not result in significant discontinuation rates. The high adherence rates in the trials suggest that the overall tolerability of Olo/Mom is acceptable. Moreover, an observational study conducted in Australia suggested patients who used a Olo/Mom nasal spray were more satisfied with the treatment’s sensory attributes and their overall experience than those who used an azelastine/fluticasone spray (Aze/Flu). Specifically, the Olo/Mom users reported significantly fewer issues with taste and smell and higher overall satisfaction (Total Satisfaction Index: ∼68 vs. 63 with Aze/Flu, p < 0.001). This suggests that Olo/Mom FDC may have a tolerability advantage, in aspects such as sensory effects, which have traditionally been a challenge for INAH

The lack of any significant systemic AEs (no HPA axis effects, no sedation, etc.) in our data is notable, possibly reflecting the systemic bioavailability of intranasal mometasone and olopatadine. Moreover, the safety profile of Olo/Mom is comparable to those of long-established therapies such as fluticasone propionate nasal spray or azelastine nasal spray used alone and is consistent with previous reports on short- and long-term use of Olo/Mom.

The efficacy and usability of Olo/Mom in routine clinical practice has been externally validated in a real-world study (2022–2023) conducted in Russia.^29^

Overall, Olo/Mom can be considered a well-tolerated treatment for SAR in adolescents and adults, with generally mild and localized adverse events.

### Limitations

Although this pooled analysis has a large sample size and provides integrated results, it cannot substitute for a single large trial and is subject to certain limitations. First, while being similar in design, the three analyzed trials are not identical. For example, the phase II study additionally included once-daily treatment arms (not analyzed in the present study) and slightly different endpoints, such as physician-assessed nasal scores. Second, we assumed the homogeneity of the treatment effects across studies. Third, while the duration of the efficacy studies (2-weeks) is relatively short. this is commonly used in SAR trials to capture peak season effects. Finally, while the POC safety study was conducted in an EEC setting, which may not fully reflect typical outpatient use conditions, it provides controlled evidence of onset of action and adds to the safety dataset. Despite these limitations, the pooled approach adopted in this study offers a robust confirmation of the benefits and risks of Olo/Mom. Moreover, the consistency of findings across multiple trials and real-world studies supports our findings.

## ORCID ID

Luis Felipe Barreiras Carbone: 0000-0003-2440-9823

Roger Hereward Gordonsmith: 0009-0003-8625-3593

Wen Wu: 0009-0005-4678-4183

Hanmant Barkate: 0000-0002-7053-584X

Wilma Terezinha Anselmo-Lima: 0000-0003-1427-2165

## Conclusion

This pooled analysis demonstrated that Olo/Mom is superior to placebo and its monotherapy constituents in reducing SAR symptoms over 14-days. Moreover, the results showed that Olo/Mom has a rapid onset of action (by 15-minutes), and administration of the treatment twice daily significantly improved RQLQ compared to the placebo. These findings, which complement observations of a previous real-world study,^29^ indicate that Olo/Mom (BID) is an effective and well-tolerated treatment option for SAR in patients aged 12-years or older. The data from these studies collectively provide a comprehensive overview of the performance of Olo/Mom in both controlled and real-world settings.

## Funding

This article was commissioned by Global Medical Affairs, Glenmark Pharmaceuticals, which provided financial support for manuscript preparation. No grant number is associated with this work.

## Declaration of competing interest

Nakanishi M received an honorarium from Global Medical Affairs, Glenmark Pharmaceuticals for the writing of this commissioned article. Carbone LFB, Aggarwal V, Gordonsmith RH, Wu W and Barkate H are employees of Glenmark Pharmaceuticals. Anselmo-Lima WT declare no conflict of interest.
