# Supplementary material for: Olopatadine plus mometasone for seasonal allergic rhinitis treatment: A pooled analysis of clinical trials
Source: Braz J Otorhinolaryngol. 2026 Apr 14;92(4):101817. doi: 10.1016/j.bjorl.2026.101817 (PMC13096916; doi:10.1016/j.bjorl.2026.101817)
Supplement: Supplementary file 1 [file mmc1.docx]

**BJORL-D-25-00368**

**Supplementary File**

**Supplementary Table S1** Description of clinical efficacy studies.

**Supplementary Table S2** Summary of ANCOVA analysis results of average a.m. and p.m. rTNSS over the 14-day treatment period (all pooled subjects, full analysis set).

**Supplementary Table S3** Summary of the repeated measures analysis results of average a.m. rTNSS over the 14-day treatment period (all pooled subjects, full analysis set).

**Supplementary Table S4** Summary of the Repeated measures analysis results of average p.m. rTNSS over the 14-day treatment period (all pooled subjects, full analysis set).

**Supplementary Table S5** Summary of the ANCOVA results of the individual domains of RQLQ(S) score on day-15.

**Supplementary Table S6** Summary of repeated measures analysis results of iTNSS onset of action.

**Supplementary Table S7** Subgroup analysis (age group, sex, race, and ethnicity): Summary of repeated measures analysis results of average a.m. and p.m. rTNSS over the 14-day treatment period (all pooled subjects, full analysis set).

**Supplementary Table S8** Summary of Treatment Emergent Adverse Events (TEAE) by system organ class, preferred term, and by relationship to study medication.

**Supplementary Table S9** Summary of Treatment Emergent Adverse Events (TEAE) by system organ class, preferred term and leading to discontinuation from the study drug.

**Supplementary Table S10** Summary of Treatment Emergent Serious Adverse Events (SAEs) overall and by system organ class and preferred term and by severity.
