# Supplementary material for: Olopatadine plus mometasone for seasonal allergic rhinitis treatment: A pooled analysis of clinical trials
Source: Braz J Otorhinolaryngol. 2026 Apr 14;92(4):101817. doi: 10.1016/j.bjorl.2026.101817 (PMC13096916; doi:10.1016/j.bjorl.2026.101817)
Supplement: Supplementary file 2 [file mmc2.pdf]

SUPPLEMENTARY FILE

Supplementary Table S1: Description of Clinical Efficacy Studies

Study ID/ NCT No. (Reference)	Number of Study Centers Location(s)	Study start Enrollment status, Date Total Enrollment / Enrollment goal	Design Control Type	Study & Ctrl Drugs Dose, Route & Regimen	Study Objective	Number of Participants by Arm randomized/ completed	Duration	Sex M/F Median Age (Range)	Diagnosis Inclusion Criteria	Primary Endpoint(s)
**NCT03444506 (GSP301-POC)** [1]	1 center<br><br>Canada	First subject enrolled: 27-Jan-2014<br><br>Last subject last visit: 28-Feb-2014<br><br>Completed<br><br>180/180	Randomized, double-blind, double-dummy, parallel-group, comparative EEC study	GSP301 (olopatadine HCl 665 µg and mometasone furoate 25 µg at 2 sprays per nostril BID)<br><br>GSP301-1 QD (olopatadine HCl 665 µg and mometasone furoate 50 µg at 2 sprays per nostril QD)<br><br>DYMISTA (azelastine hydrochloride 137 µg and fluticasone propionate 50 µg at 1 spray per nostril BID)<br><br>PATANASE (olopatadine HCl 665 µg at 2 sprays per nostril BID)<br><br>GSP301 placebo	Efficacy, safety and tolerability	GSP301 – 36/36<br><br>GSP301-1 QD – 36/35<br><br>DYMISTA – 36/34<br><br>PATANASE – 36/36<br><br>GSP301 placebo– 36/35	14 days	M/F = 91/89<br><br>Median age = 42.5 (18-65)	SAR<br><br>Male and female subjects aged 18 to 65 years, with a clinical history (for at least 2 years) of allergic rhinitis with a positive skin prick test to ragweed allergen, who provided written consent, fulfilled all the inclusion criteria and the minimum qualifying iTNSS of 6 out of 12, including a score of at least 2 for nasal congestion (not necessarily in the same diary entry) in the first 6 hours of EEC session	iTNSS change from Baseline (GSP301 and GSP301-1 QD versus placebo)
**NCT02318303 (GSP301-201)** [2]	10 centers<br><br>United States	First subject enrolled: 05 Dec 2014<br><br>Last subject last visit: 23 Feb 2015	Randomized double-blind, double-dummy, parallel-group, placebo- and active-	GSP301-1 (QD: olopatadine HCl 665 µg / mometasone furoate 50 µg; 2 sprays per nostril)	Efficacy, safety and tolerability	GSP301-1 (QD) – 158/155 subjects	14 days	M/F = 371/740<br><br>Median age = 44.6 (12.1-78.7)	SAR<br><br>Male or female subjects in general good health, 12 years of age and older, with a history of SAR to	Change from baseline in average a.m. and p.m. subject-reported rTNSS over the 14-day

		Completed<br><br>1111/1106	controlled study in subjects (12 years and older) with SAR – mountain cedar pollen	GSP301 (BID: olopatadine HCl 665 µg / mometasone furoate 25 µg; 2 sprays per nostril)<br><br>Mometasone furoate (QD 50 µg; 2 sprays per nostril)<br><br>Mometasone furoate (BID 25 µg; 2 sprays per nostril)<br><br>Olopatadine HCl (QD 665 µg; 2 sprays per nostril)<br><br>Olopatadine HCl (BID 665 µg; 2 sprays per nostril)<br><br>GSP301 placebo (2 sprays per nostril)		GSP301 (BID) – 157/152 subjects<br><br>Mometasone furoate (QD) – 160/157 subjects<br><br>Mometasone furoate (BID) – 159/153 subjects<br><br>Olopatadine HCl (QD) – 158/155 subjects<br><br>Olopatadine HCl (BID) – 160/158 subjects<br><br>GSP301 Placebo– 159/155 subjects			mountain cedar pollen for a minimum of 2 years immediately preceding the study and a positive skin prick test to mountain cedar pollen, and a 12-hour rTNSS ≥8 out of a possible 12 and a congestion score of ≥2 for the a.m. assessment	treatment period.
**NCT02631551 (GSP301-301)** [3]	37 centers<br><br>United States	First subject enrolled: 18-Mar-2016<br><br>Last subject last visit: 22-Jul- 2016<br><br>Completed<br><br>1180/1176	Randomized double-blind, placebo and active controlled, parallel-group study in subjects (12 years and older) with SAR – spring pollen (tree and grass) model	GSP301 (BID: olopatadine HCl 665 µg / mometasone furoate 25 µg; 2 sprays per nostril)<br><br>GSP301 placebo (BID; 2 sprays per nostril)<br><br>Olopatadine HCl	Efficacy, safety and tolerability	GSP301– 302/289 subjects<br><br>GSP301 placebo– 287/276 subjects<br><br>Olopatadine HCl– 297/278 subjects	14 days	M/F = 418/762<br><br>Median age = 40.0 (12-87)	SAR<br><br>Male or female subjects in general good health, 12 years of age and older, with a history of SAR to relevant seasonal allergen during spring allergy season for a minimum of 2 years immediately preceding the	The change from baseline in average a.m. and p.m. subject-reported 12-hour rTNSS over the 14-day treatment period

				(665 µg BID; 2 sprays per nostril)<br><br>Mometasone furoate (25 µg BID; 2 sprays per nostril)		Mometasone furoate–294/283 subjects			study and a positive skin prick test to allergen relevant to spring season, and a 12-hour rTNSS ≥8 out of a possible 12 and a congestion score of ≥2 for the a.m. assessment	
**NCT02870205 (GSP301-304)** [4]	43 centers<br><br>United States	First subject enrolled: 22-Aug-2016<br><br>Last subject last visit: 31-Jan-2017<br><br>Completed<br><br>1176/1176	Randomized double-blind, placebo and active controlled, parallel-group study in subjects (12 years and older) with SAR – fall or mountain cedar model	GSP301 (BID: olopatadine HCl 665 µg / mometasone furoate 25 µg; 2 sprays per nostril)<br><br>GSP301 placebo (BID; 2 sprays per nostril)<br><br>Olopatadine HCl (BID 665 µg; 2 sprays per nostril)<br><br>Mometasone furoate (BID 25 µg; 2 sprays per nostril)	Efficacy, safety and tolerability	GSP301–294/289 subjects<br><br>GSP301 placebo–294/284 subjects<br><br>Olopatadine HCL–294/287 subjects<br><br>Mometasone furoate–294/287 subjects	14 days	M/F = 435/737<br><br>Median age = 39.0 (12-82)	SAR<br><br>Male or female subjects in general good health, 12 years of age and older, with a history of SAR to relevant seasonal allergen during fall or mountain cedar season for a minimum of 2 years immediately preceding the study and a positive skin prick test to allergen relevant to the fall or mountain cedar season, and a 12-hour rTNSS ≥8 out of a possible 12 and a congestion score of ≥2 for the a.m. assessment	The change from baseline in average a.m. and p.m. subject-reported 12-hour rTNSS over the 14-day treatment period

a.m. = morning; BID = twice daily; ECG = electrocardiogram; EEC = environmental exposure chamber; ENT = ears, nose and throat; F = female; HCl = hydrochloride; iTNSS= instantaneous total nasal symptom score; M = male; PAR = perennial allergic rhinitis; p.m. = evening; POC = proof of concept; QD = once daily; rTNSS = reflective total nasal symptom score; SAR = seasonal allergic rhinitis; TEAE = treatment-emergent adverse event.

Supplementary Table S2: Summary of ANCOVA Analysis Results of Average a.m. and p.m. rTNSS over the 14-Day Treatment Period (All Pooled Subjects, Full Analysis Set)

	n		LS Mean		Comparison Between TRT1 and TRT2		
**Treatment Group Comparison (TRT1 vs. TRT2)**	**TRT1**	**TRT2**	**TRT1**	**TRT2**	**LS Mean Difference (SE)**	**95% CI**	**p Value**
GSP301 vs. Placebo	747	731	-3.46	-2.32	-1.14 (0.128)	(-1.47, -0.81)	<0.0001[a]
GSP301 vs. Olopatadine	747	744	-3.46	-2.87	-0.60 (0.127)	(-0.92, -0.27)	<0.0001[a]
GSP301 vs. Mometasone	747	746	-3.46	-2.94	-0.53 (0.127)	(-0.85, -0.20)	<0.0001[a]
Olopatadine vs. Placebo	744	731	-2.87	-2.32	-0.54 (0.128)	(-0.87, -0.22)	<0.0001[a]
Mometasone Placebo	746	731	-2.94	-2.32	-0.62 (0.127)	(-0.94, -0.29)	<0.0001[a]

a.m. = morning; ANCOVA = analysis of covariance; CI= confidence interval; LS = least square; n = number of subjects with data available; p.m. = evening; rTNSS = reflective total nasal symptom score; SE = standard error; TRT = Treatment.

[a]Statistically significant difference ($p<0.05$).

Statistical analysis model: ANCOVA with change from baseline as dependent variable, treatment group and site as fixed effect, and baseline as covariate.

Supplementary Table S3: Summary of the Repeated Measures Analysis Results of Average a.m. rTNSS over the 14-Day Treatment Period (All Pooled Subjects, Full Analysis Set)

	n		LS mean		Comparison between TRT1 and TRT2		
Treatment Group Comparison (TRT1 vs. TRT2)	**TRT1**	**TRT2**	**TRT1**	**TRT2**	**LS mean difference (SE)**	**95% CI**	**P Value**
GSP301 vs. Placebo	747	732	-3.38	-2.37	-1.00 (0.124)	(-1.25, -0.76)	<0.0001[a]
GSP301 vs. Olopatadine HCl	747	744	-3.38	-2.92	-0.46 (0.123)	(-0.70, -0.22)	0.0002[a]
GSP301 vs. Mometasone furoate	747	746	-3.38	-2.91	-0.47 (0.123)	(-0.71, -0.23)	0.0001[a]
Olopatadine HCl vs. Placebo	744	732	-2.92	-2.37	-0.55 (0.123)	(-0.79, -0.31)	<0.0001[a]
Mometasone furoate vs. Placebo	746	732	-2.91	-2.37	-0.54 (0.123)	(-0.78, -0.30)	<0.0001[a]

a.m. = morning; LS = least-squares; n = number of subjects with data available; rTNSS = reflective total nasal symptom score; SE = standard error; TRT = Treatment. [a] Statistically significant difference (p<0.05). For average a.m. rTNSS, baseline score is derived as the mean of the last 4 a.m. values prior to randomization.
Statistical analysis model: Mixed-effect model for repeated measures with change from baseline as the dependent variable, treatment group, study and site as fixed effects, baseline score as covariate and study day as the within-subject effect; variance covariance matrix used is Unstructured.

Supplementary Table S4: Summary of the Repeated Measures Analysis Results of Average p.m. rTNSS over the 14-Day Treatment Period (All Pooled Subjects, Full Analysis Set)

	n		LS mean		Comparison between TRT1 and TRT2		
**Treatment Group Comparison (TRT1 vs. TRT2)**	**TRT1**	**TRT2**	**TRT1**	**TRT2**	**LS mean difference (SE)**	**95% CI**	**P Value**
GSP301 vs. Placebo	747	732	-3.31	-2.34	-0.97 (0.122)	(-1.21, 0.73)	<0.0001[a]
GSP301 vs. Olopatadine HCl	747	744	-3.31	-2.94	-0.38 (0.121)	(-0.61, -0.14)	0.0019[a]
GSP301 vs. Mometasone furoate	747	746	-3.31	-2.88	-0.43 (0.121)	(-0.66, -0.19)	0.0004[a]
Olopatadine HCl vs. Placebo	744	732	-2.94	-2.34	-0.60 (0.122)	(-0.83, -0.36)	<0.0001[a]
Mometasone furoate vs. Placebo	746	732	-2.88	-2.34	-0.54 (0.121)	(-0.78, -0.31)	<0.0001[a]

CI = confidence interval; HCl = hydrochloride; LS = least-squares; n = number of subjects with data available; p.m. = evening; rTNSS = reflective total nasal symptom score; SE = standard error; TRT = Treatment. [a] Statistically significant difference ($p<0.05$). For average p.m. rTNSS, baseline score is derived as the mean of the last 4 p.m. values prior to randomization.

Statistical analysis model: Mixed-effect model for repeated measures with change from baseline as the dependent variable, treatment group, study and site as fixed effects, baseline score as covariate and study day as the within-subject effect; variance covariance matrix used is Unstructured.

Supplementary Table S5: Summary of the ANCOVA Results of the Individual Domains of RQLQ(S) Score on Day 15

ACTIVITIES

	n		LS mean		Comparison between TRT1 and TRT2			
**Treatment Group Comparison (TRT1 vs. TRT2)**	**TRT1**	**TRT2**	**TRT1**	**TRT2**	**LS mean difference**	**SE of LS Mean Difference**	**95% CI**	**P Value**
GSP301 vs. Placebo	735	717	-1.61	-1.06	-0.55	0.077	(-0.75, -0.35)	<0.0001*
GSP301 vs. Olopatadine HCl	735	734	-1.61	-1.29	-0.33	0.077	(-0.52, -0.13)	<0.0001*
GSP301 vs. Mometasone furoate	735	739	-1.61	-1.37	-0.25	0.076	(-0.44, -0.05)	0.0013*
Olopatadine HCl vs. Placebo	734	717	-1.29	-1.06	-0.22	0.077	(-0.42, -0.02)	0.0039*
Mometasone furoate vs. Placebo	739	717	-1.37	-1.06	-0.30	0.077	(-0.50, -0.11)	<0.0001*

EMOTIONAL

	n		LS mean		Comparison between TRT1 and TRT2			
**Treatment Group Comparison (TRT1 vs. TRT2)**	**TRT1**	**TRT2**	**TRT1**	**TRT2**	**LS mean difference**	**SE of LS Mean Difference**	**95% CI**	**P Value**
GSP301 vs. Placebo	736	718	-1.61	-1.12	-0.49	0.077	(-0.69, -0.29)	<0.0001*
GSP301 vs. Olopatadine HCl	736	733	-1.61	-1.28	-0.33	0.077	(-0.53, -0.13)	<0.0001*
GSP301 vs. mometasone Furoate	736	739	-1.61	-1.42	-0.20	0.077	(-0.39, 0.00)	0.0109*
Olopatadine HCl vs. Placebo	733	718	-1.28	-1.12	-0.16	0.077	(-0.36, 0.04)	0.0384*
Mometasone furoate vs. Placebo	739	718	-1.42	-1.12	-0.29	0.077	(-0.49, -0.10)	0.0001*

EYE SYMPTOMS

	n		LS mean		Comparison between TRT1 and TRT2			
**Treatment Group Comparison (TRT1 vs. TRT2)**	**TRT1**	**TRT2**	**TRT1**	**TRT2**	**LS mean difference**	**SE of LS Mean Difference**	**95% CI**	**P Value**
GSP301 vs. Placebo	736	718	-1.68	-1.21	-0.48	0.080	(-0.68, -0.27)	<0.0001*
GSP301 vs. Olopatadine HCl	736	735	-1.68	-1.46	-0.23	0.079	(-0.43, -0.02)	0.0040*
GSP301 vs. Mometasone furoate	736	739	-1.68	-1.44	-0.25	0.079	(-0.45, -0.05)	0.0016*
Olopatadine HCl vs. Placebo	735	718	-1.46	-1.21	-0.25	0.079	(-0.45, -0.05)	0.0016*
Mometasone furoate vs. Placebo	739	718	-1.44	-1.21	-0.23	0.079	(-0.43, -0.03)	0.0039*

NASAL SYMPTOMS

	n		LS mean		Comparison between TRT1 and TRT2			
**Treatment Group Comparison (TRT1 vs. TRT2)**	**TRT1**	**TRT2**	**TRT1**	**TRT2**	**LS mean difference**	**SE of LS Mean Difference**	**95% CI**	**P Value**
GSP301 vs. Placebo	733	717	-1.90	-1.20	-0.69	0.078	(-0.89, -0.49)	<0.0001*
GSP301 vs. Olopatadine HCl	733	735	-1.90	-1.43	-0.47	0.077	(-0.67, -0.27)	<0.0001*
GSP301 vs. Mometasone furoate	733	739	-1.90	-1.65	-0.24	0.077	(-0.44, -0.05)	0.0015*
Olopatadine HCl vs. Placebo	735	717	-1.43	-1.20	-0.22	0.078	(-0.42, -0.02)	0.0044*
Mometasone furoate vs. Placebo	739	717	-1.65	-1.20	-0.45	0.077	(-0.65, -0.25)	<0.0001*

NON-NOSE/EYE SYMPTOMS

	n		LS mean		Comparison between TRT1 and TRT2			
**Treatment Group Comparison (TRT1 vs. TRT2)**	**TRT1**	**TRT2**	**TRT1**	**TRT2**	**LS mean difference**	**SE of LS Mean Difference**	**95% CI**	**P Value**
GSP301 vs. Placebo	730	709	-1.34	-1.01	-0.33	0.074	(-0.51, -0.14)	<0.0001*
GSP301 vs. Olopatadine HCl	730	724	-1.34	-1.12	-0.22	0.073	(-0.40, -0.03)	0.0030*
GSP301 vs. Mometasone furoate	730	728	-1.34	-1.18	-0.15	0.073	(-0.34, 0.03)	0.0335*
Olopatadine HCl vs. Placebo	724	709	-1.12	-1.01	-0.11	0.074	(-0.30, 0.08)	0.1391
Mometasone furoate vs. Placebo	728	709	-1.18	-1.01	-0.17	0.073	(-0.36, 0.02)	0.0200*

PRACTICAL PROBLEMS

	n		LS mean		Comparison between TRT1 and TRT2			
**Treatment Group Comparison (TRT1 vs. TRT2)**	**TRT1**	**TRT2**	**TRT1**	**TRT2**	**LS mean difference**	**SE of LS Mean Difference**	**95% CI**	**P Value**
GSP301 vs. Placebo	735	718	-1.83	-1.21	-0.62	0.081	(-0.83, -0.41)	<0.0001*
GSP301 vs. Olopatadine HCl	735	735	-1.83	-1.54	-0.29	0.080	(-0.50, -0.08)	0.0003*
GSP301 vs. Mometasone furoate	735	740	-1.83	-1.57	-0.26	0.080	(-0.47, -0.06)	0.0011*
Olopatadine HCl vs. Placebo	735	718	-1.54	-1.21	-0.33	0.081	(-0.54, -0.12)	<0.0001*
Mometasone furoate vs. Placebo	740	718	-1.57	-1.21	-0.35	0.081	(-0.56, -0.15)	<0.0001*
**SLEEP**								
	**n**		**LS mean**		**Comparison between TRT1 and TRT2**			
**Treatment Group Comparison (TRT1 vs. TRT2)**	**TRT1**	**TRT2**	**TRT1**	**TRT2**	**LS mean difference**	**SE of LS Mean Difference**	**95% CI**	**P Value**
GSP301 vs. Placebo	736	718	-1.51	-1.11	-0.40	0.080	(-0.60, -0.19)	<0.0001*
GSP301 vs. Olopatadine HCl	736	733	-1.51	-1.28	-0.23	0.080	(-0.43, -0.02)	0.0044*
GSP301 vs. Mometasone furoate	736	740	-1.51	-1.40	-0.11	0.080	(-0.31, 0.10)	0.1714
Olopatadine HCl vs. Placebo	733	718	-1.28	-1.11	-0.17	0.080	(-0.38, 0.04)	0.0343*
Mometasone furoate vs. Placebo	740	718	-1.40	-1.11	-0.29	0.080	(-0.49, -0.08)	0.0003*

Note: Baseline is defined as the time point pre-dosing at the Randomization Visit (Visit 2). n= number of subjects with data available; LS = least squares; SE = standard error.
Statistical Analysis model: analysis of covariance (ANCOVA) with change from baseline as dependent variable, treatment group, study and site as fixed effect and baseline as covariate. Individual statistical analysis models were made separately for each of the RQLQ(S) domains. * p-value <0.05. For study GSP301-201, RQLQ (S) data was not collected for 12-17 years subgroup, according to the protocol

Supplementary Table S6: Summary of Repeated Measures Analysis Results of iTNSS Onset of Action

Treatment Group	Time Point (1)	n Active	n Placebo	L Smean Active	L Smean Placebo	LSmean Difference	95% CI	P-values Active vs.. Placebo
GSP301 (N=747)	15 minutes	741	730	-1.18	-0.95	-0.23	(-0.41, -0.05)	0.0110*
	30 minutes	741	729	-1.90	-1.46	-0.43	(-0.64, -0.23)	<0.0001*
	45 minutes	741	730	-2.49	-1.90	-0.59	(-0.82, -0.35)	<0.0001*
	60 minutes	739	726	-2.91	-2.30	-0.61	(-0.86, -0.36)	<0.0001*
	90 minutes	738	726	-3.24	-2.58	-0.66	(-0.92, -0.39)	<0.0001*
	120 minutes	737	727	-3.62	-2.89	-0.73	(-1.01, -0.46)	<0.0001*
	150 minutes	736	726	-3.86	-3.13	-0.73	(-1.02, -0.45)	<0.0001*
	180 minutes	739	728	-3.93	-3.27	-0.67	(-0.96, -0.38)	<0.0001*
	210 minutes	739	728	-4.12	-3.46	-0.66	(-0.96, -0.36)	<0.0001*
	240 minutes	739	730	-4.20	-3.48	-0.72	(-1.03, -0.42)	<0.0001*
Olopatadine HCl (N = 744)	15 minutes	738	730	-1.07	-0.95	-0.12	(-0.30, 0.05)	0.1746
	30 minutes	736	729	-1.83	-1.46	-0.36	(-0.57, -0.15)	0.0007*
	45 minutes	736	730	-2.51	-1.90	-0.61	(-0.84, -0.38)	<0.0001*
	60 minutes	731	726	-2.79	-2.30	-0.49	(-0.74, -0.24)	0.0001*
	90 minutes	732	726	-3.16	-2.58	-0.58	(-0.84, -0.31)	<0.0001*
	120 minutes	732	727	-3.46	-2.89	-0.57	(-0.84, -0.30)	<0.0001*
	150 minutes	734	726	-3.67	-3.13	-0.54	(-0.83, -0.25)	0.0002*
	180 minutes	735	728	-3.83	-3.27	-0.57	(-0.86, -0.28)	0.0001*
	210 minutes	737	728	-3.94	-3.46	-0.49	(-0.78, -0.19)	0.0014*
	240 minutes	736	730	-4.09	-3.48	-0.61	(-0.91, -0.30)	<0.0001*
Mometasone Furoate (N = 747)	15 minutes	742	730	-0.94	-0.95	0.01	(-0.17, 0.19)	0.8986
	30 minutes	742	729	-1.58	-1.46	-0.12	(-0.33, 0.09)	0.2590
	45 minutes	743	730	-2.03	-1.90	-0.13	(-0.36, 0.10)	0.2651
	60 minutes	742	726	-2.35	-2.30	-0.05	(-0.30, 0.20)	0.6817
	90 minutes	741	726	-2.74	-2.58	-0.16	(-0.43, 0.10)	0.2208
	120 minutes	742	727	-3.05	-2.89	-0.17	(-0.44, 0.10)	0.2284
	150 minutes	742	726	-3.39	-3.13	-0.26	(-0.54, 0.02)	0.0731
	180 minutes	740	728	-3.44	-3.27	-0.17	(-0.46, 0.11)	0.2364
	210 minutes	741	728	-3.66	-3.46	-0.20	(-0.50, 0.10)	0.1871
	240 minutes	740	730	-3.84	-3.48	-0.36	(-0.67, -0.06)	0.0196*

Note: Baseline is defined as the pre-dose time point at the Randomization Visit (Visit 2). n= number of subjects with data available; LS = least squares; SE = standard error.
Statistical Analysis model: Mixed-effect repeated measures model with change from baseline as the dependent variable, treatment group and site as fixed effects, baseline score as covariate, time as the within-subject effect and treatment*time interaction; variance covariance matrix Unstructured. [1] Timepoint was baseline, 15 to 240 minutes (15±3, 30±3, 45±3, 60±5, 90±5, 120±10, 150±10, 180±10, 210±10, and 240±10 minutes). * = p-value<0.05

Supplementary Table S7: Subgroup Analysis (Age Group, Sex, Race, and Ethnicity): Summary of Repeated Measures Analysis Results of Average a.m. and p.m. rTNSS over the 14-Day Treatment Period (All Pooled Subjects, Full Analysis Set)

**Treatment Comparison** (TRT1 vs. TRT2)	**Subjects (n) (TRT1 vs. TRT2)**<br>**LS Mean Difference (95% CIs)**<br>**p Value**		
	**AGE GROUP**		
	**12-17 years (N=245)**	**18-64 years (N=2581)**	**≥65 years (N=145)**
GSP301 vs. Placebo	65 vs. 62<br>-0.80 (-1.69, 0.08)<br>p=0.0731	649 vs. 633<br>-0.99 (-1.24, -0.73)<br>**p<0.0001**[a]	33 vs. 36<br>-0.79 (-1.88, 0.30)<br>p=0.1553
GSP301 vs. Olopatadine HCl	65 vs. 54<br>0.21 (-0.69, 1.12)<br>p=0.6421	649 vs. 658<br>-0.42 (-0.67, -0.17)<br>**p=0.0011**[a]	33 vs. 32<br>0.08 (-1.15, 1.31)<br>p=0.8996
GSP301 vs. Mometasone furoate	65 vs. 64<br>-0.77 (-1.60, 0.06)<br>p=0.0680	649 vs. 638<br>-0.42 (-0.67, -0.17)<br>**p=0.0011**[a]	33 vs. 44<br>0.33 (-0.77, 1.43)<br>p=0.5577
Olopatadine HCl vs. Placebo	54 vs. 62<br>-1.02 (-1.93, -0.11)<br>**p=0.0286**[a]	658 vs. 633<br>-0.57 (-0.82, -0.32)<br>**p<0.0001**[a]	32 vs. 36<br>-0.87 (-2.04, 0.31)<br>p=0.1464
Mometasone furoate vs. Placebo	64 vs. 62<br>-0.04 (-0.90, 0.83)<br>**p=0.9358**	638 vs. 633<br>-0.56 (-0.82, -0.31)<br>**p<0.0001**[a]	44 vs. 36<br>-1.11 (-2.18, -0.05)<br>**p=0.0397**[a]

	**SEX**	
	**Males (N=1062)**	**Females (N=1909)**
GSP301 vs. Placebo	235 vs. 267<br>-1.13 (-1.51, -0.76)<br>**p<0.0001**[a]	512 vs. 464<br>-0.94 (-1.20, -0.68)<br>**p<0.0001**[a]
GSP301 vs. Olopatadine HCl	235 vs. 277<br>-0.60 (-0.97, -0.22)<br>**p=0.0018**[a]	512 vs. 467<br>-0.40 (-0.66, -0.15)<br>**p=0.0021**[a]
GSP301 vs. Mometasone furoate	235 vs. 283<br>-0.60 (-0.97, -0.22)<br>**p=0.0017**[a]	512 vs. 463<br>-0.33 (-0.59, -0.08)<br>**p=0.0111**[a]

Olopatadine HCl vs. Placebo	277 vs. 267<br>-0.54 (-0.90, -0.17)<br>**p=0.0038**[a]	467 vs. 464<br>-0.53 (-0.80, -0.27)<br>**p<0.0001**[a]
Mometasone furoate vs. Placebo	283 vs. 267<br>-0.54 (-0.90, -0.18)<br>**p=0.0036**[a]	463 vs. 464<br>-0.60 (-0.87, -0.34)<br>**p<0.0001**[a]

RACE

	White<br>(N=2381)	Black/African American<br>(N=503)	Other<br>(N=87)
GSP301 vs.<br>Placebo	617 vs. 589<br>-1.28 (-1.55, -1.01)<br>**p<0.0001**[a]	108 vs. 128<br>-0.43 (-1.08, 0.23)<br>p=0.2037	22 vs. 14<br>-3.33 (-7.14, 0.48)<br>p=0.0850
GSP301 vs.<br>Olopatadine HCl	617 vs. 591<br>-0.67 (-0.94, -0.40)<br>**p<0.0001**[a]	108 vs. 130<br>-0.34 (-0.99, 0.32)<br>p=0.3122	22 vs. 23<br>1.28 (-1.45, 4.00)<br>p=0.3508
GSP301 vs.<br>Mometasone furoate	617 vs. 582<br>-0.51 (-0.78, -0.23)<br>**p=0.0003**[a]	108 vs. 136<br>-0.6 (-1.33, -0.03)<br>**p=0.0392**[a]	22 vs. 28<br>0.47 (-2.17, 3.10)<br>p=0.7223
Olopatadine HCl vs.<br>Placebo	591 vs. 589<br>-0.61 (-0.88, -0.33)<br>**p<0.0001**[a]	130 vs. 128<br>-0.09 (-0.72, 0.54)<br>p=0.7841	23 vs. 14<br>-4.61 (-8.64, -0.58)<br>**p=0.0259**[a]
Mometasone fumarate vs. Placebo	582 vs. 589<br>-0.77 (-1.05, -0.49)<br>**p<0.0001**[a]	136 vs. 128<br>0.25 (-0.37, 0.88)<br>p=0.4259	28 vs. 14<br>-3.80 (-7.28, -0.32)<br>**p=0.0328**[a]

ETHNICITY

	Hispanic or Latino<br>(N=879)	Not Hispanic or Latino<br>(N=2092)
GSP301 vs.<br>Placebo	218 vs. 202<br>-0.97 (-1.42, -0.51)<br>**p<0.0001**[a]	529 vs. 529<br>-1.07 (-1.36, -0.78)<br>**p<0.0001**[a]
GSP301 vs.<br>Olopatadine HCl	218 vs. 235<br>-0.52 (0.96, -0.08)<br>**p=0.0203**[a]	529 vs. 509<br>-0.46 (-0.75, -0.17)<br>**p=0.0019**[a]
GSP301 vs.<br>Mometasone furoate	218 vs. 224<br>-0.09 (-0.53, 0.36)<br>p=0.6968	529 vs. 522<br>-0.5 (-0.87, -0.29)<br>**p<0.0001**[a]
Olopatadine HCl vs.<br>Placebo	235 vs. 202<br>-0.45 (-0.89, 0.00)<br>**p=0.0499**[a]	509 vs. 529<br>-0.61 (-0.90, -0.31)<br>**p<0.0001**[a]

Mometasone furoate vs. Placebo	224 vs. 202<br>-0.88 (-1.33, -0.43)<br>**p=0.0001[a]**	522 vs. 529<br>-0.49 (-0.78, -0.20)<br>**p=0.0010[a]**

a.m. = morning; CI = confidence interval; HCl = hydrochloride; LS = least-squares; n = number of subjects in the treatment group with data available; N = number of subjects in the age subgroup; p.m. = evening; rTNSS = reflective total nasal symptom score; TRT: Treatment. **Error! Reference source not found.**Statistically significant difference (p<0.05).

Supplementary Table S8: Summary of Treatment Emergent Adverse Events (TEAE) by System Organ Class, Preferred Term, and by Relationship to Study Medication

SYSTEM ORGAN CLASS Preferred Term	TREATMENT GROUP: GSP301 Placebo BID (N=776) [a]		GSP301 BID (N=789) [a]		Olopatadine HCL BID (N=751) [a]		Mometasone Furoate BID (N=746) [a]		Total (N=3062) [a]	
	Subjects n (%) (b)	Events (c) (Event Rate)	Subjects n (%) (b)	Events (c) (Event Rate)	Subjects n (%) (b)	Events (c) (Event Rate)	Subjects n (%) (b)	Events (c) (Event Rate)	Subjects n (%) (b)	Events (c) (Event Rate)
Total	74 (9.5)	93 (7.0)	110 (13.9)	135 (7.0)	99 (13.2)	133 (7.3)	59 (7.9)	84 (7.5)	342 (11.2)	445 (7.2)
Related	20 (2.6)	26 (2.0)	51 (6.5)	59 (3.1)	45 (6.0)	51 (2.8)	20 (2.7)	31 (2.8)	136 (4.4)	167 (2.7)
BLOOD AND LYMPHATIC SYSTEM DISORDERS	0	0	0	0	1 (0.1)	1 (0.1)	0	0	1 (0.0)	1 (0.0)
Related	0	0	0	0	0	0	0	0	0	0
Anemia	0	0	0	0	1 (0.1)	1 (0.1)	0	0	1 (0.0)	1 (0.0)
Related	0	0	0	0	0	0	0	0	0	0
CARDIAC DISORDERS	0	0	0	0	0	0	1 (0.1)	1 (0.1)	1 (0.0)	1 (0.0)
Related	0	0	0	0	0	0	0	0	0	0
Supraventricular extrasystoles	0	0	0	0	0	0	1 (0.1)	1 (0.1)	1 (0.0)	1 (0.0)
Related	0	0	0	0	0	0	0	0	0	0
EAR AND LABYRINTH DISORDERS	3 (0.4)	3 (0.2)	2 (0.3)	2 (0.1)	2 (0.3)	2 (0.1)	1 (0.1)	1 (0.1)	8 (0.3)	8 (0.1)
Related	0	0	0	0	0	0	0	0	0	0
Ear pain	2 (0.3)	2 (0.2)	2 (0.3)	2 (0.1)	2 (0.3)	2 (0.1)	1 (0.1)	1 (0.1)	7 (0.2)	7 (0.1)
Related	0	0	0	0	0	0	0	0	0	0
Vertigo	1 (0.1)	1 (0.1)	0	0	0	0	0	0	1 (0.0)	1 (0.0)
Related	0	0	0	0	0	0	0	0	0	0
EYE DISORDERS	1 (0.1)	1 (0.1)	1 (0.1)	1 (0.1)	0	0	4 (0.5)	6 (0.5)	6 (0.2)	8 (0.1)
Related	0	0	0	0	0	0	0	0	0	0
Blepharitis	1 (0.1)	1 (0.1)	0	0	0	0	0	0	1 (0.0)	1 (0.0)
Related	0	0	0	0	0	0	0	0	0	0
Conjunctival hemorrhage	0	0	1 (0.1)	1 (0.1)	0	0	0	0	1 (0.0)	1 (0.0)
Related	0	0	0	0	0	0	0	0	0	0

Eye pain	0	0	0	0	0	0	2 (0.3)	2 (0.2)	2 (0.1)	2 (0.0)
Related	0	0	0	0	0	0	0	0	0	0
Eye pruritus	0	0	0	0	0	0	1 (0.1)	1 (0.1)	1 (0.0)	1 (0.0)
Related	0	0	0	0	0	0	0	0	0	0
Ocular hyperemia	0	0	0	0	0	0	2 (0.3)	2 (0.2)	2 (0.1)	2 (0.0)
Related	0	0	0	0	0	0	0	0	0	0
Pterygium	0	0	0	0	0	0	1 (0.1)	1 (0.1)	1 (0.0)	1 (0.0)
Related	0	0	0	0	0	0	0	0	0	0
GASTROINTESTINAL DISORDERS	5 (0.6)	5 (0.4)	9 (1.1)	9 (0.5)	8 (1.1)	13 (0.7)	3 (0.4)	3 (0.3)	25 (0.8)	30 (0.5)
Related	0	0	3 (0.4)	3 (0.2)	3 (0.4)	3 (0.2)	2 (0.3)	2 (0.2)	8 (0.3)	8 (0.1)
Abdominal discomfort	0	0	2 (0.3)	2 (0.1)	0	0	0	0	2 (0.1)	2 (0.0)
Related	0	0	0	0	0	0	0	0	0	0
Abdominal distension	1 (0.1)	1 (0.1)	0	0	0	0	0	0	1 (0.0)	1 (0.0)
Related	0	0	0	0	0	0	0	0	0	0
Abdominal pain	0	0	1 (0.1)	1 (0.1)	0	0	0	0	1 (0.0)	1 (0.0)
Related	0	0	1 (0.1)	1 (0.1)	0	0	0	0	1 (0.0)	1 (0.0)
Abdominal pain upper	0	0	2 (0.3)	2 (0.1)	0	0	0	0	2 (0.1)	2 (0.0)
Related	0	0	1 (0.1)	1 (0.1)	0	0	0	0	1 (0.0)	1 (0.0)
Constipation	0	0	0	0	1 (0.1)	1 (0.1)	0	0	1 (0.0)	1 (0.0)
Related	0	0	0	0	0	0	0	0	0	0
Diarrhea	1 (0.1)	1 (0.1)	2 (0.3)	2 (0.1)	4 (0.5)	4 (0.2)	0	0	7 (0.2)	7 (0.1)
Related	0	0	0	0	0	0	0	0	0	0
Dry mouth	0	0	1 (0.1)	1 (0.1)	1 (0.1)	1 (0.1)	2 (0.3)	2 (0.2)	4 (0.1)	4 (0.1)
Related	0	0	1 (0.1)	1 (0.1)	1 (0.1)	1 (0.1)	1 (0.1)	1 (0.1)	3 (0.1)	3 (0.0)
Dyspepsia	0	0	1 (0.1)	1 (0.1)	0	0	0	0	1 (0.0)	1 (0.0)
Related	0	0	0	0	0	0	0	0	0	0
Flatulence	0	0	0	0	1 (0.1)	1 (0.1)	0	0	1 (0.0)	1 (0.0)
Related	0	0	0	0	0	0	0	0	0	0
Large intestinal obstruction	0	0	0	0	1 (0.1)	1 (0.1)	0	0	1 (0.0)	1 (0.0)
Related	0	0	0	0	0	0	0	0	0	0
Large intestine perforation	0	0	0	0	1 (0.1)	1 (0.1)	0	0	1 (0.0)	1 (0.0)
Related	0	0	0	0	0	0	0	0	0	0
Nausea	1 (0.1)	1 (0.1)	0	0	2 (0.3)	2 (0.1)	1 (0.1)	1 (0.1)	4 (0.1)	4 (0.1)
Related	0	0	0	0	2 (0.3)	2 (0.1)	1 (0.1)	1 (0.1)	3 (0.1)	3 (0.0)
Stomatitis	0	0	0	0	1 (0.1)	1 (0.1)	0	0	1 (0.0)	1 (0.0)
Related	0	0	0	0	0	0	0	0	0	0
Toothache	2 (0.3)	2 (0.2)	0	0	0	0	0	0	2 (0.1)	2 (0.0)
Related	0	0	0	0	0	0	0	0	0	0
Vomiting	0	0	0	0	1 (0.1)	1 (0.1)	0	0	1 (0.0)	1 (0.0)
Related	0	0	0	0	0	0	0	0	0	0
GENERAL DISORDERS AND ADMINISTRATION SITE CONDITIONS	3 (0.4)	3 (0.2)	4 (0.5)	5 (0.3)	5 (0.7)	6 (0.3)	2 (0.3)	4 (0.4)	14 (0.5)	18 (0.3)

Related	2 (0.3)	2 (0.2)	2 (0.3)	2 (0.1)	2 (0.3)	2 (0.1)	2 (0.3)	4 (0.4)	8 (0.3)	10 (0.2)
Chest pain	0	0	0	0	1 (0.1)	1 (0.1)	1 (0.1)	1 (0.1)	2 (0.1)	2 (0.0)
Related	0	0	0	0	0	0	1 (0.1)	1 (0.1)	1 (0.0)	1 (0.0)
Chills	0	0	0	0	0	0	1 (0.1)	1 (0.1)	1 (0.0)	1 (0.0)
Related	0	0	0	0	0	0	1 (0.1)	1 (0.1)	1 (0.0)	1 (0.0)
Fatigue	1 (0.1)	1 (0.1)	2 (0.3)	2 (0.1)	2 (0.3)	2 (0.1)	1 (0.1)	1 (0.1)	6 (0.2)	6 (0.1)
Related	1 (0.1)	1 (0.1)	2 (0.3)	2 (0.1)	1 (0.1)	1 (0.1)	1 (0.1)	1 (0.1)	5 (0.2)	5 (0.1)
Feeling jittery	1 (0.1)	1 (0.1)	0	0	0	0	0	0	1 (0.0)	1 (0.0)
Related	1 (0.1)	1 (0.1)	0	0	0	0	0	0	1 (0.0)	1 (0.0)
Mucosal erosion	0	0	0	0	1 (0.1)	1 (0.1)	0	0	1 (0.0)	1 (0.0)
Related	0	0	0	0	1 (0.1)	1 (0.1)	0	0	1 (0.0)	1 (0.0)
Pain	1 (0.1)	1 (0.1)	0	0	1 (0.1)	1 (0.1)	0	0	2 (0.1)	2 (0.0)
Related	0	0	0	0	0	0	0	0	0	0
Pyrexia	0	0	1 (0.1)	1 (0.1)	1 (0.1)	1 (0.1)	0	0	2 (0.1)	2 (0.0)
Related	0	0	0	0	0	0	0	0	0	0
Thirst	0	0	0	0	0	0	1 (0.1)	1 (0.1)	1 (0.0)	1 (0.0)
Related	0	0	0	0	0	0	1 (0.1)	1 (0.1)	1 (0.0)	1 (0.0)
Vessel puncture site bruise	0	0	1 (0.1)	1 (0.1)	0	0	0	0	1 (0.0)	1 (0.0)
Related	0	0	0	0	0	0	0	0	0	0
Vessel puncture site hematoma	0	0	1 (0.1)	1 (0.1)	0	0	0	0	1 (0.0)	1 (0.0)
Related	0	0	0	0	0	0	0	0	0	0
IMMUNE SYSTEM DISORDERS	1 (0.1)	1 (0.1)	1 (0.1)	1 (0.1)	0	0	0	0	2 (0.1)	2 (0.0)
Related	0	0	0	0	0	0	0	0	0	0
Seasonal allergy	1 (0.1)	1 (0.1)	1 (0.1)	1 (0.1)	0	0	0	0	2 (0.1)	2 (0.0)
Related	0	0	0	0	0	0	0	0	0	0
INFECTIONS AND INFESTATIONS	11 (1.4)	11 (0.8)	21 (2.7)	22 (1.1)	24 (3.2)	26 (1.4)	14 (1.9)	16 (1.4)	70 (2.3)	75 (1.2)
Related	0	0	0	0	1 (0.1)	1 (0.1)	0	0	1 (0.0)	1 (0.0)
Acute sinusitis	1 (0.1)	1 (0.1)	0	0	1 (0.1)	1 (0.1)	0	0	2 (0.1)	2 (0.0)
Related	0	0	0	0	0	0	0	0	0	0
Bacterial infection	0	0	1 (0.1)	1 (0.1)	0	0	0	0	1 (0.0)	1 (0.0)
Related	0	0	0	0	0	0	0	0	0	0
Bronchitis	0	0	0	0	2 (0.3)	2 (0.1)	1 (0.1)	1 (0.1)	3 (0.1)	3 (0.0)
Related	0	0	0	0	0	0	0	0	0	0
Candida infection	0	0	0	0	1 (0.1)	1 (0.1)	0	0	1 (0.0)	1 (0.0)
Related	0	0	0	0	1 (0.1)	1 (0.1)	0	0	1 (0.0)	1 (0.0)
Cystitis	0	0	0	0	1 (0.1)	1 (0.1)	0	0	1 (0.0)	1 (0.0)
Related	0	0	0	0	0	0	0	0	0	0
Diverticulitis	0	0	0	0	1 (0.1)	1 (0.1)	0	0	1 (0.0)	1 (0.0)
Related	0	0	0	0	0	0	0	0	0	0
Epididymitis	0	0	1 (0.1)	1 (0.1)	0	0	0	0	1 (0.0)	1 (0.0)
Related	0	0	0	0	0	0	0	0	0	0
Gastroenteritis	1 (0.1)	1 (0.1)	1 (0.1)	1 (0.1)	1 (0.1)	1 (0.1)	1 (0.1)	1 (0.1)	4 (0.1)	4 (0.1)
Related	0	0	0	0	0	0	0	0	0	0

Gastroenteritis viral	0	0	0	0	0	0	1 (0.1)	1 (0.1)	1 (0.0)	1 (0.0)
Related	0	0	0	0	0	0	0	0	0	0
Hordeolum	0	0	0	0	1 (0.1)	1 (0.1)	1 (0.1)	1 (0.1)	2 (0.1)	2 (0.0)
Related	0	0	0	0	0	0	0	0	0	0
Lower respiratory tract infection	0	0	1 (0.1)	1 (0.1)	0	0	0	0	1 (0.0)	1 (0.0)
Related	0	0	0	0	0	0	0	0	0	0
Onychomycosis	0	0	0	0	1 (0.1)	1 (0.1)	0	0	1 (0.0)	1 (0.0)
Related	0	0	0	0	0	0	0	0	0	0
Oral candidiasis	1 (0.1)	1 (0.1)	0	0	0	0	0	0	1 (0.0)	1 (0.0)
Related	0	0	0	0	0	0	0	0	0	0
Oral herpes	2 (0.3)	2 (0.2)	0	0	2 (0.3)	2 (0.1)	0	0	4 (0.1)	4 (0.1)
Related	0	0	0	0	0	0	0	0	0	0
Osteomyelitis	1 (0.1)	1 (0.1)	0	0	0	0	0	0	1 (0.0)	1 (0.0)
Related	0	0	0	0	0	0	0	0	0	0
Otitis externa	0	0	0	0	1 (0.1)	1 (0.1)	0	0	1 (0.0)	1 (0.0)
Related	0	0	0	0	0	0	0	0	0	0
Otitis media	0	0	1 (0.1)	1 (0.1)	2 (0.3)	2 (0.1)	2 (0.3)	2 (0.2)	5 (0.2)	5 (0.1)
Related	0	0	0	0	0	0	0	0	0	0
Otitis media acute	0	0	0	0	0	0	1 (0.1)	1 (0.1)	1 (0.0)	1 (0.0)
Related	0	0	0	0	0	0	0	0	0	0
Peritonsillar abscess	0	0	0	0	0	0	1 (0.1)	1 (0.1)	1 (0.0)	1 (0.0)
Related	0	0	0	0	0	0	0	0	0	0
Pharyngitis	0	0	1 (0.1)	1 (0.1)	0	0	0	0	1 (0.0)	1 (0.0)
Related	0	0	0	0	0	0	0	0	0	0
Pharyngitis streptococcal	0	0	0	0	1 (0.1)	1 (0.1)	1 (0.1)	1 (0.1)	2 (0.1)	2 (0.0)
Related	0	0	0	0	0	0	0	0	0	0
Pyuria	0	0	2 (0.3)	2 (0.1)	0	0	0	0	2 (0.1)	2 (0.0)
Related	0	0	0	0	0	0	0	0	0	0
Respiratory tract infection	0	0	1 (0.1)	1 (0.1)	0	0	0	0	1 (0.0)	1 (0.0)
Related	0	0	0	0	0	0	0	0	0	0
Respiratory tract infection viral	0	0	1 (0.1)	1 (0.1)	0	0	0	0	1 (0.0)	1 (0.0)
Related	0	0	0	0	0	0	0	0	0	0
Sinusitis	0	0	1 (0.1)	1 (0.1)	1 (0.1)	1 (0.1)	0	0	2 (0.1)	2 (0.0)
Related	0	0	0	0	0	0	0	0	0	0
Tonsillitis	0	0	0	0	1 (0.1)	1 (0.1)	0	0	1 (0.0)	1 (0.0)
Related	0	0	0	0	0	0	0	0	0	0
Tonsillitis streptococcal	0	0	0	0	0	0	1 (0.1)	1 (0.1)	1 (0.0)	1 (0.0)
Related	0	0	0	0	0	0	0	0	0	0
Tooth abscess	0	0	0	0	1 (0.1)	1 (0.1)	0	0	1 (0.0)	1 (0.0)
Related	0	0	0	0	0	0	0	0	0	0
Tracheitis	0	0	0	0	0	0	1 (0.1)	1 (0.1)	1 (0.0)	1 (0.0)
Related	0	0	0	0	0	0	0	0	0	0
Upper respiratory tract infection	2 (0.3)	2 (0.2)	3 (0.4)	3 (0.2)	4 (0.5)	4 (0.2)	3 (0.4)	3 (0.3)	12 (0.4)	12 (0.2)

Related	0	0	0	0	0	0	0	0	0	0
Urinary tract infection	2 (0.3)	2 (0.2)	4 (0.5)	4 (0.2)	2 (0.3)	2 (0.1)	1 (0.1)	1 (0.1)	9 (0.3)	9 (0.1)
Related	0	0	0	0	0	0	0	0	0	0
Viral infection	0	0	1 (0.1)	1 (0.1)	0	0	0	0	1 (0.0)	1 (0.0)
Related	0	0	0	0	0	0	0	0	0	0
Viral upper respiratory tract infection	1 (0.1)	1 (0.1)	3 (0.4)	3 (0.2)	2 (0.3)	2 (0.1)	1 (0.1)	1 (0.1)	7 (0.2)	7 (0.1)
Related	0	0	0	0	0	0	0	0	0	0
INJURY, POISONING AND PROCEDURAL COMPLICATIONS	5 (0.6)	6 (0.5)	6 (0.8)	6 (0.3)	3 (0.4)	3 (0.2)	3 (0.4)	3 (0.3)	17 (0.6)	18 (0.3)
Related	0	0	0	0	0	0	0	0	0	0
Animal scratch	0	0	0	0	0	0	1 (0.1)	1 (0.1)	1 (0.0)	1 (0.0)
Related	0	0	0	0	0	0	0	0	0	0
Arthropod bite	0	0	1 (0.1)	1 (0.1)	1 (0.1)	1 (0.1)	0	0	2 (0.1)	2 (0.0)
Related	0	0	0	0	0	0	0	0	0	0
Contusion	0	0	1 (0.1)	1 (0.1)	0	0	0	0	1 (0.0)	1 (0.0)
Related	0	0	0	0	0	0	0	0	0	0
Eye injury	0	0	0	0	1 (0.1)	1 (0.1)	1 (0.1)	1 (0.1)	2 (0.1)	2 (0.0)
Related	0	0	0	0	0	0	0	0	0	0
Face injury	0	0	1 (0.1)	1 (0.1)	0	0	0	0	1 (0.0)	1 (0.0)
Related	0	0	0	0	0	0	0	0	0	0
Fall	1 (0.1)	1 (0.1)	0	0	0	0	0	0	1 (0.0)	1 (0.0)
Related	0	0	0	0	0	0	0	0	0	0
Foot fracture	1 (0.1)	1 (0.1)	0	0	0	0	0	0	1 (0.0)	1 (0.0)
Related	0	0	0	0	0	0	0	0	0	0
Foreign body	1 (0.1)	1 (0.1)	0	0	0	0	0	0	1 (0.0)	1 (0.0)
Related	0	0	0	0	0	0	0	0	0	0
Laceration	1 (0.1)	1 (0.1)	1 (0.1)	1 (0.1)	0	0	0	0	2 (0.1)	2 (0.0)
Related	0	0	0	0	0	0	0	0	0	0
Ligament sprain	0	0	2 (0.3)	2 (0.1)	0	0	1 (0.1)	1 (0.1)	3 (0.1)	3 (0.0)
Related	0	0	0	0	0	0	0	0	0	0
Nasal injury	0	0	0	0	1 (0.1)	1 (0.1)	0	0	1 (0.0)	1 (0.0)
Related	0	0	0	0	0	0	0	0	0	0
Procedural pain	1 (0.1)	1 (0.1)	0	0	0	0	0	0	1 (0.0)	1 (0.0)
Related	0	0	0	0	0	0	0	0	0	0
Tooth fracture	1 (0.1)	1 (0.1)	0	0	0	0	0	0	1 (0.0)	1 (0.0)
Related	0	0	0	0	0	0	0	0	0	0
INVESTIGATIONS	5 (0.6)	6 (0.5)	1 (0.1)	1 (0.1)	4 (0.5)	5 (0.3)	3 (0.4)	7 (0.6)	13 (0.4)	19 (0.3)
Related	1 (0.1)	1 (0.1)	0	0	2 (0.3)	2 (0.1)	1 (0.1)	3 (0.3)	4 (0.1)	6 (0.1)
Alanine aminotransferase increased	1 (0.1)	1 (0.1)	0	0	2 (0.3)	2 (0.1)	1 (0.1)	1 (0.1)	4 (0.1)	4 (0.1)
Related	0	0	0	0	1 (0.1)	1 (0.1)	1 (0.1)	1 (0.1)	2 (0.1)	2 (0.0)
Aspartate aminotransferase increased	1 (0.1)	1 (0.1)	0	0	1 (0.1)	1 (0.1)	1 (0.1)	1 (0.1)	3 (0.1)	3 (0.0)

Related	0	0	0	0	0	0	1 (0.1)	1 (0.1)	1 (0.0)	1 (0.0)
Blood alkaline phosphatase increased	0	0	0	0	0	0	1 (0.1)	1 (0.1)	1 (0.0)	1 (0.0)
Related	0	0	0	0	0	0	1 (0.1)	1 (0.1)	1 (0.0)	1 (0.0)
Blood bilirubin increased	0	0	0	0	1 (0.1)	1 (0.1)	0	0	1 (0.0)	1 (0.0)
Related	0	0	0	0	1 (0.1)	1 (0.1)	0	0	1 (0.0)	1 (0.0)
Blood creatine phosphokinase	0	0	0	0	1 (0.1)	1 (0.1)	0	0	1 (0.0)	1 (0.0)
Related	0	0	0	0	0	0	0	0	0	0
Blood creatinine increased	0	0	0	0	0	0	1 (0.1)	1 (0.1)	1 (0.0)	1 (0.0)
Related	0	0	0	0	0	0	0	0	0	0
Blood glucose increased	1 (0.1)	1 (0.1)	0	0	0	0	0	0	1 (0.0)	1 (0.0)
Related	0	0	0	0	0	0	0	0	0	0
Blood potassium increased	0	0	0	0	0	0	1 (0.1)	1 (0.1)	1 (0.0)	1 (0.0)
Related	0	0	0	0	0	0	0	0	0	0
Blood urea increased	0	0	0	0	0	0	1 (0.1)	1 (0.1)	1 (0.0)	1 (0.0)
Related	0	0	0	0	0	0	0	0	0	0
Cardiac murmur	0	0	0	0	0	0	1 (0.1)	1 (0.1)	1 (0.0)	1 (0.0)
Related	0	0	0	0	0	0	0	0	0	0
Gamma-glutamyltransferase	1 (0.1)	1 (0.1)	0	0	0	0	0	0	1 (0.0)	1 (0.0)
Related	0	0	0	0	0	0	0	0	0	0
Hepatic enzyme increased	2 (0.3)	2 (0.2)	0	0	0	0	0	0	2 (0.1)	2 (0.0)
Related	1 (0.1)	1 (0.1)	0	0	0	0	0	0	1 (0.0)	1 (0.0)
Liver function test increased	0	0	1 (0.1)	1 (0.1)	0	0	0	0	1 (0.0)	1 (0.0)
Related	0	0	0	0	0	0	0	0	0	0
METABOLISM AND NUTRITION DISORDERS	0	0	3 (0.4)	3 (0.2)	1 (0.1)	1 (0.1)	0	0	4 (0.1)	4 (0.1)
Related	0	0	0	0	1 (0.1)	1 (0.1)	0	0	1 (0.0)	1 (0.0)
Hyperglycemia	0	0	1 (0.1)	1 (0.1)	0	0	0	0	1 (0.0)	1 (0.0)
Related	0	0	0	0	0	0	0	0	0	0
Increased appetite	0	0	1 (0.1)	1 (0.1)	1 (0.1)	1 (0.1)	0	0	2 (0.1)	2 (0.0)
Related	0	0	0	0	1 (0.1)	1 (0.1)	0	0	1 (0.0)	1 (0.0)
Vitamin D deficiency	0	0	1 (0.1)	1 (0.1)	0	0	0	0	1 (0.0)	1 (0.0)
Related	0	0	0	0	0	0	0	0	0	0
MUSCULOSKELETAL AND CONNECTIVE TISSUE DISORDERS	3 (0.4)	3 (0.2)	6 (0.8)	8 (0.4)	3 (0.4)	3 (0.2)	3 (0.4)	3 (0.3)	15 (0.5)	17 (0.3)
Related	0	0	0	0	0	0	0	0	0	0
Arthralgia	1 (0.1)	1 (0.1)	0	0	1 (0.1)	1 (0.1)	0	0	2 (0.1)	2 (0.0)
Related	0	0	0	0	0	0	0	0	0	0
Back pain	1 (0.1)	1 (0.1)	4 (0.5)	4 (0.2)	1 (0.1)	1 (0.1)	0	0	6 (0.2)	6 (0.1)
Related	0	0	0	0	0	0	0	0	0	0
Costochondritis	0	0	1 (0.1)	1 (0.1)	0	0	0	0	1 (0.0)	1 (0.0)

Related	0	0	0	0	0	0	0	0	0	0
Joint swelling	0	0	1 (0.1)	1 (0.1)	0	0	0	0	1 (0.0)	1 (0.0)
Related	0	0	0	0	0	0	0	0	0	0
Musculoskeletal pain	0	0	0	0	0	0	1 (0.1)	1 (0.1)	1 (0.0)	1 (0.0)
Related	0	0	0	0	0	0	0	0	0	0
Neck pain	0	0	1 (0.1)	1 (0.1)	0	0	1 (0.1)	1 (0.1)	2 (0.1)	2 (0.0)
Related	0	0	0	0	0	0	0	0	0	0
Pain in extremity	0	0	1 (0.1)	1 (0.1)	1 (0.1)	1 (0.1)	0	0	2 (0.1)	2 (0.0)
Related	0	0	0	0	0	0	0	0	0	0
Pain in jaw	0	0	0	0	0	0	1 (0.1)	1 (0.1)	1 (0.0)	1 (0.0)
Related	0	0	0	0	0	0	0	0	0	0
Tendonitis	1 (0.1)	1 (0.1)	0	0	0	0	0	0	1 (0.0)	1 (0.0)
Related	0	0	0	0	0	0	0	0	0	0
NEOPLASMS BENIGN, MALIGNANT AND UNSPECIFIED (INCL CYSTS AND POLYPS)	1 (0.1)	1 (0.1)	0	0	0	0	0	0	1 (0.0)	1 (0.0)
Related	0	0	0	0	0	0	0	0	0	0
Basal cell carcinoma	1 (0.1)	1 (0.1)	0	0	0	0	0	0	1 (0.0)	1 (0.0)
Related	0	0	0	0	0	0	0	0	0	0
NERVOUS SYSTEM DISORDERS	17 (2.2)	20 (1.5)	35 (4.4)	36 (1.9)	32 (4.3)	35 (1.9)	12 (1.6)	19 (1.7)	96 (3.1)	110 (1.8)
Related	8 (1.0)	11 (0.8)	33 (4.2)	34 (1.8)	21 (2.8)	21 (1.1)	6 (0.8)	11 (1.0)	68 (2.2)	77 (1.2)
Dizziness	0	0	1 (0.1)	2 (0.1)	0	0	2 (0.3)	2 (0.2)	3 (0.1)	4 (0.1)
Related	0	0	1 (0.1)	2 (0.1)	0	0	1 (0.1)	1 (0.1)	2 (0.1)	3 (0.0)
Dysgeusia	2 (0.3)	3 (0.2)	24 (3.0)	24 (1.3)	16 (2.1)	16 (0.9)	0	0	42 (1.4)	43 (0.7)
Related	2 (0.3)	3 (0.2)	24 (3.0)	24 (1.3)	16 (2.1)	16 (0.9)	0	0	42 (1.4)	43 (0.7)
Headache	14 (1.8)	16 (1.2)	6 (0.8)	6 (0.3)	8 (1.1)	8 (0.4)	7 (0.9)	11 (1.0)	35 (1.1)	41 (0.7)
Related	6 (0.8)	8 (0.6)	4 (0.5)	4 (0.2)	2 (0.3)	2 (0.1)	3 (0.4)	6 (0.5)	15 (0.5)	20 (0.3)
Hypergeusia	0	0	1 (0.1)	1 (0.1)	0	0	0	0	1 (0.0)	1 (0.0)
Related	0	0	1 (0.1)	1 (0.1)	0	0	0	0	1 (0.0)	1 (0.0)
Hypogeusia	0	0	0	0	1 (0.1)	1 (0.1)	0	0	1 (0.0)	1 (0.0)
Related	0	0	0	0	1 (0.1)	1 (0.1)	0	0	1 (0.0)	1 (0.0)
Ischemic stroke	0	0	0	0	1 (0.1)	1 (0.1)	0	0	1 (0.0)	1 (0.0)
Related	0	0	0	0	0	0	0	0	0	0
Lethargy	0	0	1 (0.1)	1 (0.1)	0	0	0	0	1 (0.0)	1 (0.0)
Related	0	0	1 (0.1)	1 (0.1)	0	0	0	0	1 (0.0)	1 (0.0)
Migraine	0	0	0	0	2 (0.3)	3 (0.2)	0	0	2 (0.1)	3 (0.0)
Related	0	0	0	0	0	0	0	0	0	0
Nerve compression	0	0	0	0	1 (0.1)	1 (0.1)	0	0	1 (0.0)	1 (0.0)
Related	0	0	0	0	0	0	0	0	0	0
Seizure	0	0	0	0	1 (0.1)	1 (0.1)	0	0	1 (0.0)	1 (0.0)
Related	0	0	0	0	1 (0.1)	1 (0.1)	0	0	1 (0.0)	1 (0.0)
Sinus headache	0	0	0	0	2 (0.3)	2 (0.1)	2 (0.3)	2 (0.2)	4 (0.1)	4 (0.1)
Related	0	0	0	0	0	0	0	0	0	0

Somnolence	0	0	2 (0.3)	2 (0.1)	2 (0.3)	2 (0.1)	2 (0.3)	4 (0.4)	6 (0.2)	8 (0.1)
Related	0	0	2 (0.3)	2 (0.1)	1 (0.1)	1 (0.1)	2 (0.3)	4 (0.4)	5 (0.2)	7 (0.1)
Syncope	1 (0.1)	1 (0.1)	0	0	0	0	0	0	1 (0.0)	1 (0.0)
Related	0	0	0	0	0	0	0	0	0	0
PREGNANCY, PUERPERIUM AND PERINATAL CONDITIONS	0	0	1 (0.1)	1 (0.1)	0	0	0	0	1 (0.0)	1 (0.0)
Related	0	0	0	0	0	0	0	0	0	0
Abortion spontaneous	0	0	1 (0.1)	1 (0.1)	0	0	0	0	1 (0.0)	1 (0.0)
Related	0	0	0	0	0	0	0	0	0	0
PSYCHIATRIC DISORDERS	0	0	2 (0.3)	2 (0.1)	1 (0.1)	1 (0.1)	1 (0.1)	1 (0.1)	4 (0.1)	4 (0.1)
Related	0	0	1 (0.1)	1 (0.1)	1 (0.1)	1 (0.1)	1 (0.1)	1 (0.1)	3 (0.1)	3 (0.0)
Anxiety	0	0	1 (0.1)	1 (0.1)	0	0	0	0	1 (0.0)	1 (0.0)
Related	0	0	0	0	0	0	0	0	0	0
Insomnia	0	0	1 (0.1)	1 (0.1)	0	0	1 (0.1)	1 (0.1)	2 (0.1)	2 (0.0)
Related	0	0	1 (0.1)	1 (0.1)	0	0	1 (0.1)	1 (0.1)	2 (0.1)	2 (0.0)
Irritability	0	0	0	0	1 (0.1)	1 (0.1)	0	0	1 (0.0)	1 (0.0)
Related	0	0	0	0	1 (0.1)	1 (0.1)	0	0	1 (0.0)	1 (0.0)
RENAL AND URINARY DISORDERS	1 (0.1)	1 (0.1)	0	0	0	0	1 (0.1)	1 (0.1)	2 (0.1)	2 (0.0)
Related	0	0	0	0	0	0	0	0	0	0
Dysuria	0	0	0	0	0	0	1 (0.1)	1 (0.1)	1 (0.0)	1 (0.0)
Related	0	0	0	0	0	0	0	0	0	0
Hematuria	1 (0.1)	1 (0.1)	0	0	0	0	0	0	1 (0.0)	1 (0.0)
Related	0	0	0	0	0	0	0	0	0	0
REPRODUCTIVE SYSTEM AND BREAST DISORDERS	2 (0.3)	2 (0.2)	1 (0.1)	1 (0.1)	1 (0.1)	1 (0.1)	0	0	4 (0.1)	4 (0.1)
Related	0	0	0	0	0	0	0	0	0	0
Dysmenorrhea	1 (0.1)	1 (0.1)	0	0	1 (0.1)	1 (0.1)	0	0	2 (0.1)	2 (0.0)
Related	0	0	0	0	0	0	0	0	0	0
Ovarian cyst	1 (0.1)	1 (0.1)	0	0	0	0	0	0	1 (0.0)	1 (0.0)
Related	0	0	0	0	0	0	0	0	0	0
Prostatitis	0	0	1 (0.1)	1 (0.1)	0	0	0	0	1 (0.0)	1 (0.0)
Related	0	0	0	0	0	0	0	0	0	0
RESPIRATORY, THORACIC AND MEDIASTINAL DISORDERS	24 (3.1)	27 (2.0)	30 (3.8)	33 (1.7)	26 (3.5)	30 (1.6)	15 (2.0)	15 (1.3)	95 (3.1)	105 (1.7)
Related	10 (1.3)	12 (0.9)	17 (2.2)	19 (1.0)	17 (2.3)	19 (1.0)	10 (1.3)	10 (0.9)	54 (1.8)	60 (1.0)
Asthma	0	0	2 (0.3)	2 (0.1)	2 (0.3)	2 (0.1)	0	0	4 (0.1)	4 (0.1)
Related	0	0	0	0	0	0	0	0	0	0
Cough	3 (0.4)	3 (0.2)	5 (0.6)	5 (0.3)	2 (0.3)	2 (0.1)	0	0	10 (0.3)	10 (0.2)
Related	0	0	0	0	0	0	0	0	0	0
Dry throat	0	0	0	0	0	0	1 (0.1)	1 (0.1)	1 (0.0)	1 (0.0)
Related	0	0	0	0	0	0	1 (0.1)	1 (0.1)	1 (0.0)	1 (0.0)
Dysphonia	1 (0.1)	1 (0.1)	0	0	0	0	0	0	1 (0.0)	1 (0.0)
Related	0	0	0	0	0	0	0	0	0	0

Epistaxis	5 (0.6)	5 (0.4)	8 (1.0)	8 (0.4)	11 (1.5)	13 (0.7)	6 (0.8)	6 (0.5)	30 (1.0)	32 (0.5)
Related	4 (0.5)	4 (0.3)	7 (0.9)	7 (0.4)	8 (1.1)	10 (0.5)	4 (0.5)	4 (0.4)	23 (0.8)	25 (0.4)
Nasal congestion	1 (0.1)	1 (0.1)	0	0	0	0	0	0	1 (0.0)	1 (0.0)
Related	0	0	0	0	0	0	0	0	0	0
Nasal discomfort	6 (0.8)	6 (0.5)	8 (1.0)	8 (0.4)	4 (0.5)	4 (0.2)	4 (0.5)	4 (0.4)	22 (0.7)	22 (0.4)
Related	6 (0.8)	6 (0.5)	6 (0.8)	6 (0.3)	4 (0.5)	4 (0.2)	4 (0.5)	4 (0.4)	20 (0.7)	20 (0.3)
Nasal dryness	0	0	1 (0.1)	1 (0.1)	3 (0.4)	3 (0.2)	2 (0.3)	2 (0.2)	6 (0.2)	6 (0.1)
Related	0	0	1 (0.1)	1 (0.1)	3 (0.4)	3 (0.2)	0	0	4 (0.1)	4 (0.1)
Nasal inflammation	0	0	1 (0.1)	1 (0.1)	0	0	1 (0.1)	1 (0.1)	2 (0.1)	2 (0.0)
Related	0	0	1 (0.1)	1 (0.1)	0	0	1 (0.1)	1 (0.1)	2 (0.1)	2 (0.0)
Nasal mucosal erosion	1 (0.1)	1 (0.1)	1 (0.1)	1 (0.1)	0	0	1 (0.1)	1 (0.1)	3 (0.1)	3 (0.0)
Related	1 (0.1)	1 (0.1)	0	0	0	0	0	0	1 (0.0)	1 (0.0)
Oropharyngeal pain	7 (0.9)	7 (0.5)	1 (0.1)	1 (0.1)	2 (0.3)	2 (0.1)	0	0	10 (0.3)	10 (0.2)
Related	0	0	1 (0.1)	1 (0.1)	0	0	0	0	1 (0.0)	1 (0.0)
Paranasal sinus discomfort	0	0	1 (0.1)	1 (0.1)	0	0	0	0	1 (0.0)	1 (0.0)
Related	0	0	0	0	0	0	0	0	0	0
Sinus congestion	1 (0.1)	1 (0.1)	0	0	1 (0.1)	1 (0.1)	0	0	2 (0.1)	2 (0.0)
Related	0	0	0	0	0	0	0	0	0	0
Sneezing	1 (0.1)	1 (0.1)	1 (0.1)	1 (0.1)	0	0	0	0	2 (0.1)	2 (0.0)
Related	0	0	1 (0.1)	1 (0.1)	0	0	0	0	1 (0.0)	1 (0.0)
Throat irritation	1 (0.1)	1 (0.1)	2 (0.3)	2 (0.1)	3 (0.4)	3 (0.2)	0	0	6 (0.2)	6 (0.1)
Related	1 (0.1)	1 (0.1)	2 (0.3)	2 (0.1)	2 (0.3)	2 (0.1)	0	0	5 (0.2)	5 (0.1)
Upper-airway cough syndrome	0	0	1 (0.1)	1 (0.1)	0	0	0	0	1 (0.0)	1 (0.0)
Related	0	0	0	0	0	0	0	0	0	0
Wheezing	0	0	1 (0.1)	1 (0.1)	0	0	0	0	1 (0.0)	1 (0.0)
Related	0	0	0	0	0	0	0	0	0	0
SKIN AND SUBCUTANEOUS TISSUE DISORDERS	3 (0.4)	3 (0.2)	4 (0.5)	4 (0.2)	5 (0.7)	5 (0.3)	4 (0.5)	4 (0.4)	16 (0.5)	16 (0.3)
Related	0	0	0	0	1 (0.1)	1 (0.1)	0	0	1 (0.0)	1 (0.0)
Acne	0	0	0	0	0	0	1 (0.1)	1 (0.1)	1 (0.0)	1 (0.0)
Related	0	0	0	0	0	0	0	0	0	0
Blister	0	0	1 (0.1)	1 (0.1)	0	0	0	0	1 (0.0)	1 (0.0)
Related	0	0	0	0	0	0	0	0	0	0
Dermatitis	0	0	0	0	1 (0.1)	1 (0.1)	0	0	1 (0.0)	1 (0.0)
Related	0	0	0	0	0	0	0	0	0	0
Dermatitis contact	0	0	1 (0.1)	1 (0.1)	0	0	0	0	1 (0.0)	1 (0.0)
Related	0	0	0	0	0	0	0	0	0	0
Hyperhidrosis	0	0	0	0	1 (0.1)	1 (0.1)	0	0	1 (0.0)	1 (0.0)
Related	0	0	0	0	1 (0.1)	1 (0.1)	0	0	1 (0.0)	1 (0.0)
Pruritus	0	0	1 (0.1)	1 (0.1)	0	0	1 (0.1)	1 (0.1)	2 (0.1)	2 (0.0)
Related	0	0	0	0	0	0	0	0	0	0
Rash	1 (0.1)	1 (0.1)	0	0	1 (0.1)	1 (0.1)	0	0	2 (0.1)	2 (0.0)
Related	0	0	0	0	0	0	0	0	0	0
Rash papular	0	0	1 (0.1)	1 (0.1)	0	0	0	0	1 (0.0)	1 (0.0)

Related	0	0	0	0	0	0	0	0	0	0
Rash pruritic	0	0	0	0	0	0	1 (0.1)	1 (0.1)	1 (0.0)	1 (0.0)
Related	0	0	0	0	0	0	0	0	0	0
Skin disorder	0	0	0	0	1 (0.1)	1 (0.1)	0	0	1 (0.0)	1 (0.0)
Related	0	0	0	0	0	0	0	0	0	0
Urticaria	2 (0.3)	2 (0.2)	0	0	1 (0.1)	1 (0.1)	1 (0.1)	1 (0.1)	4 (0.1)	4 (0.1)
Related	0	0	0	0	0	0	0	0	0	0
VASCULAR DISORDERS	0	0	0	0	1 (0.1)	1 (0.1)	0	0	1 (0.0)	1 (0.0)
Related	0	0	0	0	0	0	0	0	0	0
Vasodilatation	0	0	0	0	1 (0.1)	1 (0.1)	0	0	1 (0.0)	1 (0.0)
Related	0	0	0	0	0	0	0	0	0	0

Note: Includes subjects in Study GSP301-POC, GSP301-201, GSP301-301, GSP301-304 and have received BID treatment only.

[a] N = Total number of subjects in each treatment group in the safety analysis set.

[b] n = number of subjects with adverse events in each MedDRA term; Number (%) of subjects with AEs, sorted on international order for system organ class and alphabetically for preferred term. Percentages are based on total number of subjects in the safety set within each treatment group. At each level of summation (overall, system organ class, preferred term), subjects are only counted once. Adverse events will be classified as 'Related' to study treatment if the relationship is categorized as Possible, Probable, or Definite according to the CRF. Missing relationship to study drug will be considered as related. Adverse events were coded using MedDRA version 19.0.

[c] All events in that category; Event Rate = Number of TEAEs divided by the total duration of treatment in days across all subjects in given treatment group, multiplied by 100

Supplementary Table S9: Summary of Treatment Emergent Adverse Events (TEAE) by System Organ Class, Preferred Term and Leading to Discontinuation from the Study Drug

SYSTEM ORGAN CLASS<br>Preferred Term	GSP301 Placebo BID (N=776) [a]		GSP301 BID (N=789) [a]		Olopatadine HCL BID (N=751) [a]		Mometasone Furoate BID (N=746) [a]		Total (N=3062) [a]	
	Subjects n (%) (b)	Events (c) (Event Rate)	Subjects n (%) (b)	Events (c) (Event Rate)	Subjects n (%) (b)	Events (c) (Event Rate)	Subjects n (%) (b)	Events (c) (Event Rate)	Subjects n (%) (b)	Events (c) (Event Rate)
Total	1 (0.1)	1 (8.3)	3 (0.4)	3 (13.6)	7 (0.9)	8 (19.0)	6 (0.8)	10 (16.4)	17 (0.6)	22 (16.1)
GASTROINTESTINAL DISORDERS	0	0	0	0	1 (0.1)	1 (2.4)	0	0	1 (0.0)	1 (0.7)
Nausea	0	0	0	0	1 (0.1)	1 (2.4)	0	0	1 (0.0)	1 (0.7)
GENERAL DISORDERS AND ADMINISTRATION SITE CONDITIONS	0	0	0	0	0	0	1 (0.1)	3 (4.9)	1 (0.0)	3 (2.2)
Chest pain	0	0	0	0	0	0	1 (0.1)	1 (1.6)	1 (0.0)	1 (0.7)
Chills	0	0	0	0	0	0	1 (0.1)	1 (1.6)	1 (0.0)	1 (0.7)
Fatigue	0	0	0	0	0	0	1 (0.1)	1 (1.6)	1 (0.0)	1 (0.7)
INFECTIONS AND INFESTATIONS	0	0	3 (0.4)	3 (13.6)	5 (0.7)	5 (11.9)	4 (0.5)	5 (8.2)	12 (0.4)	13 (9.5)
Bronchitis	0	0	0	0	1 (0.1)	1 (2.4)	0	0	1 (0.0)	1 (0.7)
Otitis media	0	0	0	0	0	0	1 (0.1)	1 (1.6)	1 (0.0)	1 (0.7)
Otitis media acute	0	0	0	0	0	0	1 (0.1)	1 (1.6)	1 (0.0)	1 (0.7)
Pharyngitis	0	0	1 (0.1)	1 (4.5)	0	0	0	0	1 (0.0)	1 (0.7)
Pharyngitis streptococcal	0	0	0	0	1 (0.1)	1 (2.4)	1 (0.1)	1 (1.6)	2 (0.1)	2 (1.5)
Sinusitis	0	0	1 (0.1)	1 (4.5)	0	0	0	0	1 (0.0)	1 (0.7)
Upper respiratory tract infection	0	0	1 (0.1)	1 (4.5)	2 (0.3)	2 (4.8)	2 (0.3)	2 (3.3)	5 (0.2)	5 (3.6)
Urinary tract infection	0	0	0	0	1 (0.1)	1 (2.4)	0	0	1 (0.0)	1 (0.7)
INJURY, POISONING AND PROCEDURAL COMPLICATIONS	1 (0.1)	1 (8.3)	0	0	0	0	0	0	1 (0.0)	1 (0.7)
Foot fracture	1 (0.1)	1 (8.3)	0	0	0	0	0	0	1 (0.0)	1 (0.7)
MUSCULOSKELETAL AND CONNECTIVE TISSUE DISORDERS	0	0	0	0	0	0	1 (0.1)	1 (1.6)	1 (0.0)	1 (0.7)
Musculoskeletal pain	0	0	0	0	0	0	1 (0.1)	1 (1.6)	1 (0.0)	1 (0.7)
NERVOUS SYSTEM DISORDERS	0	0	0	0	2 (0.3)	2 (4.8)	1 (0.1)	1 (1.6)	3 (0.1)	3 (2.2)
Dizziness	0	0	0	0	0	0	1 (0.1)	1 (1.6)	1 (0.0)	1 (0.7)
Dysgeusia	0	0	0	0	1 (0.1)	1 (2.4)	0	0	1 (0.0)	1 (0.7)
Seizure	0	0	0	0	1 (0.1)	1 (2.4)	0	0	1 (0.0)	1 (0.7)

Note: Includes subjects in Study GSP301-POC, GSP301-201, GSP301-301, GSP301-304 and have received BID treatment only.
[a] N = Total number of subjects in each treatment group in the safety analysis set.
[b] n = number of subjects with adverse events in each MedDRA term; Number (%) of subjects with AEs, sorted on international order for system organ class and alphabetically for preferred term. Percentages are based on total number of subjects in the safety set within each treatment group. At each level of summation (overall, system organ class, preferred term), subjects are only counted once.
[c] All events in that category; Event Rate = Number of TEAEs divided by the total duration of treatment in days across all subjects in given treatment group, multiplied by 100. Adverse events were coded using MedDRA version 19.0.

Supplementary Table S10: Summary of Treatment Emergent Serious Adverse Events (SAEs) Overall and by System Organ Class and Preferred Term and by Severity

TREATMENT GROUP

SYSTEM ORGAN CLASS<br>Preferred Term	GSP301 Placebo BID (N=776) [a]		GSP301 BID (N=789) [a]		Olopatadine HCL BID (N=751) [a]		Mometasone Furoate BID (N=746) [a]		Total (N=3062) [a]	
	Subjects n (%) (b)	Events (c) (Event Rate)	Subjects n (%) (b)	Events (c) (Event Rate)	Subjects n (%) (b)	Events (c) (Event Rate)	Subjects n (%) (b)	Events (c) (Event Rate)	Subjects n (%) (b)	Events (c) (Event Rate)
Total (%)	1 (0.1)	3 (8.3)	1 (0.1)	1 (7.1)	2 (0.3)	4 (9.3)	1 (0.1)	1 (7.1)	5 (0.2)	9 (8.4)
GASTROINTESTINAL DISORDERS	0	0	0	0	1 (0.1)	2 (4.7)	0	0	1 (0.0)	2 (1.9)
Large intestinal obstruction	0	0	0	0	1 (0.1)	1 (2.3)	0	0	1 (0.0)	1 (0.9)
Large intestine perforation	0	0	0	0	1 (0.1)	1 (2.3)	0	0	1 (0.0)	1 (0.9)
INFECTIONS AND INFESTATIONS	1 (0.1)	1 (2.8)	0	0	1 (0.1)	1 (2.3)	1 (0.1)	1 (7.1)	3 (0.1)	3 (2.8)
Diverticulitis	0	0	0	0	1 (0.1)	1 (2.3)	0	0	1 (0.0)	1 (0.9)
Osteomyelitis	1 (0.1)	1 (2.8)	0	0	0	0	0	0	1 (0.0)	1 (0.9)
Peritonsillar abscess	0	0	0	0	0	0	1 (0.1)	1 (7.1)	1 (0.0)	1 (0.9)
INJURY, POISONING AND PROCEDURAL COMPLICATIONS	1 (0.1)	1 (2.8)	0	0	0	0	0	0	1 (0.0)	1 (0.9)
Foot fracture	1 (0.1)	1 (2.8)	0	0	0	0	0	0	1 (0.0)	1 (0.9)
NERVOUS SYSTEM DISORDERS	1 (0.1)	1 (2.8)	0	0	1 (0.1)	1 (2.3)	0	0	2 (0.1)	2 (1.9)
Ischemic stroke	0	0	0	0	1 (0.1)	1 (2.3)	0	0	1 (0.0)	1 (0.9)
Syncope	1 (0.1)	1 (2.8)	0	0	0	0	0	0	1 (0.0)	1 (0.9)
PREGNANCY, PUERPERIUM AND PERINATAL CONDITIONS	0	0	1 (0.1)	1 (7.1)	0	0	0	0	1 (0.0)	1 (0.9)
Abortion spontaneous	0	0	1 (0.1)	1 (7.1)	0	0	0	0	1 (0.0)	1 (0.9)
**BASED ON SEVERITY**										
Total (%)	1 (0.1)	3 (8.3)	1 (0.1)	1 (7.1)	2 (0.3)	4 (9.3)	1 (0.1)	1 (7.1)	5 (0.2)	9 (8.4)
Mild	0	0	0	0	0	0	0	0	0	0
Moderate	0	0	1 (0.1)	1 (7.1)	0	0	1 (0.1)	1 (7.1)	2 (0.1)	2 (1.9)
Severe	1 (0.1)	3 (8.3)	0	0	2 (0.3)	4 (9.3)	0	0	3 (0.1)	7 (6.5)
GASTROINTESTINAL DISORDERS	0	0	0	0	1 (0.1)	2 (4.7)	0	0	1 (0.0)	2 (1.9)
Mild	0	0	0	0	0	0	0	0	0	0
Moderate	0	0	0	0	0	0	0	0	0	0
Severe	0	0	0	0	1 (0.1)	2 (4.7)	0	0	1 (0.0)	2 (1.9)
Large intestinal obstruction	0	0	0	0	1 (0.1)	1 (2.3)	0	0	1 (0.0)	1 (0.9)
Mild	0	0	0	0	0	0	0	0	0	0
Moderate	0	0	0	0	0	0	0	0	0	0
Severe	0	0	0	0	1 (0.1)	1 (2.3)	0	0	1 (0.0)	1 (0.9)
Large intestine perforation	0	0	0	0	1 (0.1)	1 (2.3)	0	0	1 (0.0)	1 (0.9)
Mild	0	0	0	0	0	0	0	0	0	0

	Moderate	0	0	0	0	0	0	0	0	0	0
	Severe	0	0	0	0	1 (0.1)	1 (2.3)	0	0	1 (0.0)	1 (0.9)
INFECTIONS AND INFESTATIONS		1 (0.1)	1 (2.8)	0	0	1 (0.1)	1 (2.3)	1 (0.1)	1 (7.1)	3 (0.1)	3 (2.8)
	Mild	0	0	0	0	0	0	0	0	0	0
	Moderate	0	0	0	0	0	0	1 (0.1)	1 (7.1)	1 (0.0)	1 (0.9)
	Severe	1 (0.1)	1 (2.8)	0	0	1 (0.1)	1 (2.3)	0	0	2 (0.1)	2 (1.9)
Diverticulitis		0	0	0	0	1 (0.1)	1 (2.3)	0	0	1 (0.0)	1 (0.9)
	Mild	0	0	0	0	0	0	0	0	0	0
	Moderate	0	0	0	0	0	0	0	0	0	0
	Severe	0	0	0	0	1 (0.1)	1 (2.3)	0	0	1 (0.0)	1 (0.9)
Osteomyelitis		1 (0.1)	1 (2.8)	0	0	0	0	0	0	1 (0.0)	1 (0.9)
	Mild	0	0	0	0	0	0	0	0	0	0
	Moderate	0	0	0	0	0	0	0	0	0	0
	Severe	1 (0.1)	1 (2.8)	0	0	0	0	0	0	1 (0.0)	1 (0.9)
Peritonsillar abscess		0	0	0	0	0	0	1 (0.1)	1 (7.1)	1 (0.0)	1 (0.9)
	Mild	0	0	0	0	0	0	0	0	0	0
	Moderate	0	0	0	0	0	0	1 (0.1)	1 (7.1)	1 (0.0)	1 (0.9)
	Severe	0	0	0	0	0	0	0	0	0	0
INJURY, POISONING AND PROCEDURAL COMPLICATIONS		1 (0.1)	1 (2.8)	0	0	0	0	0	0	1 (0.0)	1 (0.9)
	Mild	0	0	0	0	0	0	0	0	0	0
	Moderate	0	0	0	0	0	0	0	0	0	0
	Severe	1 (0.1)	1 (2.8)	0	0	0	0	0	0	1 (0.0)	1 (0.9)
Foot fracture		1 (0.1)	1 (2.8)	0	0	0	0	0	0	1 (0.0)	1 (0.9)
	Mild	0	0	0	0	0	0	0	0	0	0
	Moderate	0	0	0	0	0	0	0	0	0	0
	Severe	1 (0.1)	1 (2.8)	0	0	0	0	0	0	1 (0.0)	1 (0.9)
NERVOUS SYSTEM DISORDERS		1 (0.1)	1 (2.8)	0	0	1 (0.1)	1 (2.3)	0	0	2 (0.1)	2 (1.9)
	Mild	0	0	0	0	0	0	0	0	0	0
	Moderate	0	0	0	0	0	0	0	0	0	0
	Severe	1 (0.1)	1 (2.8)	0	0	1 (0.1)	1 (2.3)	0	0	2 (0.1)	2 (1.9)
Ischemic stroke		0	0	0	0	1 (0.1)	1 (2.3)	0	0	1 (0.0)	1 (0.9)
	Mild	0	0	0	0	0	0	0	0	0	0
	Moderate	0	0	0	0	0	0	0	0	0	0
	Severe	0	0	0	0	1 (0.1)	1 (2.3)	0	0	1 (0.0)	1 (0.9)
Syncope		1 (0.1)	1 (2.8)	0	0	0	0	0	0	1 (0.0)	1 (0.9)
	Mild	0	0	0	0	0	0	0	0	0	0
	Moderate	0	0	0	0	0	0	0	0	0	0
	Severe	1 (0.1)	1 (2.8)	0	0	0	0	0	0	1 (0.0)	1 (0.9)
PREGNANCY, PUERPERIUM AND PERINATAL CONDITIONS		0	0	1 (0.1)	1 (7.1)	0	0	0	0	1 (0.0)	1 (0.9)
	Mild	0	0	0	0	0	0	0	0	0	0
	Moderate	0	0	1 (0.1)	1 (7.1)	0	0	0	0	1 (0.0)	1 (0.9)
	Severe	0	0	0	0	0	0	0	0	0	0
Abortion spontaneous		0	0	1 (0.1)	1 (7.1)	0	0	0	0	1 (0.0)	1 (0.9)
	Mild	0	0	0	0	0	0	0	0	0	0
	Moderate	0	0	1 (0.1)	1 (7.1)	0	0	0	0	1 (0.0)	1 (0.9)
	Severe	0	0	0	0	0	0	0	0	0	0

Note: Includes subjects in Study GSP301-POC, GSP301-201, GSP301-301, GSP301-304 and have received BID treatment only.
[a] N = Total number of subjects in each treatment group in the safety analysis set.
[b] n = number of subjects with adverse events in each MedDRA term; Number (%) of subjects with AEs, sorted on international order for system organ class and alphabetically for preferred term. Percentages are based on total number of subjects in the safety set within each treatment group. At each level of summation (overall, system organ class, preferred term), subjects are only counted once. If a subject has multiple occurrences of the same System Organ Class (SOC) or Preferred Term (PT), then only the most severe event will be summarized in the tables for that SOC and PT. Adverse events were coded using MedDRA version 19.0.
[c] All events in that category; Event Rate = Number of TEAEs divided by the total duration of treatment in days across all subjects in given treatment group, multiplied by 100.

REFERENCES

1. Patel P, Salapatek AM, Tantry SK. Effect of olopatadine-mometasone combination nasal spray on seasonal allergic rhinitis symptoms in an environmental exposure chamber study. Ann Allergy Asthma Immunol. 2019;122:160-6.e161.
2. Andrews CP, Mohar D, Salhi Y, Tantry SK. Efficacy and safety of twice-daily and once-daily olopatadine-mometasone combination nasal spray for seasonal allergic rhinitis. Ann Allergy Asthma Immunol. 2020;124:171-8.e172.
3. Hampel FC, Pedinoff AJ, Jacobs RL, Caracta CF, Tantry SK, et al. Olopatadine-mometasone combination nasal spray: evaluation of efficacy and safety in patients with seasonal allergic rhinitis. Allergy Asthma Proc. 2019;40:261-72.
4. Gross GN, Berman G, Amar NJ, Caracta CF, Tantry SK. Efficacy and safety of olopatadine-mometasone combination nasal spray for the treatment of seasonal allergic rhinitis. Ann Allergy Asthma Immunol. 2019;122:630-8.e633.
